# Overexpression of Differentially Expressed Genes Identified in Non-pathogenic and Pathogenic Entamoeba histolytica Clones Allow Identification of New Pathogenicity Factors Involved in Amoebic Liver Abscess Formation

**DOI:** 10.1371/journal.ppat.1005853

**Published:** 2016-08-30

**Authors:** Martin Meyer, Helena Fehling, Jenny Matthiesen, Stephan Lorenzen, Kathrin Schuldt, Hannah Bernin, Mareen Zaruba, Corinna Lender, Thomas Ernst, Harald Ittrich, Thomas Roeder, Egbert Tannich, Hannelore Lotter, Iris Bruchhaus

**Affiliations:** 1 Bernhard Nocht Institute for Tropical Medicine, Hamburg, Germany; 2 Diagnostic and Interventional Radiology Department and Clinic, University Medical Center Hamburg-Eppendorf, Hamburg, Germany; 3 Zoological Institute, Molecular Physiology, Christian-Albrechts University Kiel, Kiel, Germany; University of California Los Angeles, UNITED STATES

## Abstract

We here compared pathogenic (p) and non-pathogenic (np) isolates of *Entamoeba histolytica* to identify molecules involved in the ability of this parasite to induce amoebic liver abscess (ALA)-like lesions in two rodent models for the disease. We performed a comprehensive analysis of 12 clones (A1–A12) derived from a non-pathogenic isolate HM-1:IMSS-A and 12 clones (B1–B12) derived from a pathogenic isolate HM-1:IMSS-B. “Non-pathogenicity” included the induction of small and quickly resolved lesions while “pathogenicity” comprised larger abscess development that overstayed day 7 post infection. All A-clones were designated as non-pathogenic, whereas 4 out of 12 B-clones lost their ability to induce ALAs in gerbils. No correlation between ALA formation and cysteine peptidase (CP) activity, haemolytic activity, erythrophagocytosis, motility or cytopathic activity was found. To identify the molecular framework underlying different pathogenic phenotypes, three clones were selected for in-depth transcriptome analyses. Comparison of a non-pathogenic clone A1^np^ with pathogenic clone B2^p^ revealed 76 differentially expressed genes, whereas comparison of a non-pathogenic clone B8^np^ with B2^p^ revealed only 19 differentially expressed genes. Only six genes were found to be similarly regulated in the two non-pathogenic clones A1^np^ and B8^np^ in comparison with the pathogenic clone B2^p^. Based on these analyses, we chose 20 candidate genes and evaluated their roles in ALA formation using the respective gene-overexpressing transfectants. We conclude that different mechanisms lead to loss of pathogenicity. In total, we identified eight proteins, comprising a metallopeptidase, C2 domain proteins, alcohol dehydrogenases and hypothetical proteins, that affect the pathogenicity of *E*. *histolytica*.

## Author Summary

The pathogen *Entamoeba histolytica* can live asymptomatically in the human gut, or it can disrupt the intestinal barrier and induce life-threatening abscesses in different organs, most often in the liver. The molecular framework that enables this invasive, highly pathogenic phenotype is still not well understood. In order to identify factors that are positively or negatively correlated for invasion and destruction of the liver, we used a unique tool, *E*. *histolytica* clones that differ dramatically in their pathogenicity, while sharing almost identical genetic background. Based on comprehensive transcriptome studies of these clones, we identified a set of candidate genes that are potentially involved in pathogenicity. Using ectopic overexpression of the most promising candidates, either in pathogenic or in non-pathogenic *Entamoeba* clones, we identified genes where high expression reduced pathogenicity and only one gene that increased pathogenicity to a certain extend.

Taken together, the current study identifies novel pathogenicity factors of *E*. *histolytica* and highlights the observation that various different genes contribute to pathogenicity.

## Introduction

The protozoan parasite *Entamoeba histolytica* is responsible for approximately 50 million cases of invasive amoebiasis per year, resulting in an annual death toll of 40,000–100,000 [1]. The parasite life cycle is relatively simple, comprising infectious cysts that can survive outside the host and vegetative trophozoites that proliferate in the human gut. After infection, *E*. *histolytica* trophozoites can asymptomatically persist for months or years in its human host [2].

Under as yet unknown circumstances, *E*. *histolytica* escapes from the gut lumen, either by penetrating the intestinal mucosa and inducing colitis, or by disseminating to other organs, most commonly the liver, where it induces abscess formation. The factors that determine the clinical outcomes of *E*. *histolytica* infections are not well understood. Possible factors comprise genetic make-up of the parasite and/or host, the immune response mounted by the host, concomitant infections and host diet. Identification of *E*. *histolytica* pathogenicity factors is a major topic in the field. Recently, research dealing with *E*. *histolytica* pathogenicity factors has mainly focused on a triad of protein families, namely, galactose/N-acetyl d-galactosamine–inhibitable Gal/GalNAc-lectins, cysteine peptidases (CPs) and amoebapores. Results obtained using transgenic amoebae supported the hypothesis that these molecules are involved in amoebic liver abscess (ALA) formation [3–6]. Nevertheless, homologues of the majority of these potential pathogenicity factors are also present in the non-pathogenic sister species *Entamoeba dispar*, a commensal protozoan that is genetically closely related to *E*. *histolytica*. Therefore, it remains to be shown whether one of these factors or their combination is responsible for amoeba pathogenicity or whether additional factors are involved. Thus, the mechanisms and processes enabling *E*. *histolytica* to penetrate host tissues and induce colitis and/or liver abscesses are still not understood. One straight-forward approach of identifying pathogenicity factors is a direct comparison of pathogenic and non-pathogenic *E*. *histolytica* isolates that has been performed using comparative microarray and proteome approaches [7–10]. Unfortunately, these studies used two isolates with completely different genetic backgrounds (pathogenic isolate HM-1:IMSS and non-pathogenic isolate Rahman). This rendered the straight-forward identification of pathogenicity factors almost impossible. In addition, an in-depth phenotypical characterisation of the Rahman isolate revealed a number of genomic defects that presumably interfere with its virulence capacity [10].

Recently, we identified two cell lines that were both derived from the clinical *E*. *histolytica* isolate HM-1:IMSS but which significantly differ in their pathogenicity. Whereas cell line HM-1:IMSS-A completely lost its ability to induce ALAs in gerbils (*Meriones unguiculatus*) and mice (*Mus musculus*), cell line HM-1:IMSS-B is highly aggressive and induces large ALAs. Comparative transcriptomic and proteomic studies of these cell lines have already been performed [11]. The studies revealed 31 differentially (≥3-fold) expressed genes [12] and 31 proteins with differential abundances in HM-1:IMSS-A and HM-1:IMSS-B [11]. However, an overlap of only two molecules was found between the proteomic and transcriptomic approaches.

Until now, neither pathogenic nor non-pathogenic cell lines have been cloned, and therefore it is possible that they consist of a mixture of cells. Thus, in the present study, both cell lines were cloned to obtain homogenous cell populations, allowing analyses of the pathogenicity factors in a straight-forward fashion. Twelve clones derived from the non-pathogenic cell line HM-1:IMSS-A (A1–A12) and 12 clones derived from the pathogenic cell line HM-1:IMSS-B (B1–B12) were generated. All 24 clones were analysed for their ability to induce ALAs in gerbils and for their specific CP activity. One non-pathogenic A-clone (A1^np^), one pathogenic B-clone (B2^p^) and one non-pathogenic B-clone (B8^np^) were analysed in more detail, including their time course of ALA formation, growth, size, motility, haemolytic activity, erythrophagocytosis, cytopathic activity and transcriptome profiles. Furthermore, transfectants overexpressing genes that were identified as differentially expressed between the pathogenic and non-pathogenic clones were tested for their involvement in ALA formation.

## Results

### Generation of *E*. *histolytica* clones and pathogenicity determinations

**Fig 1:**
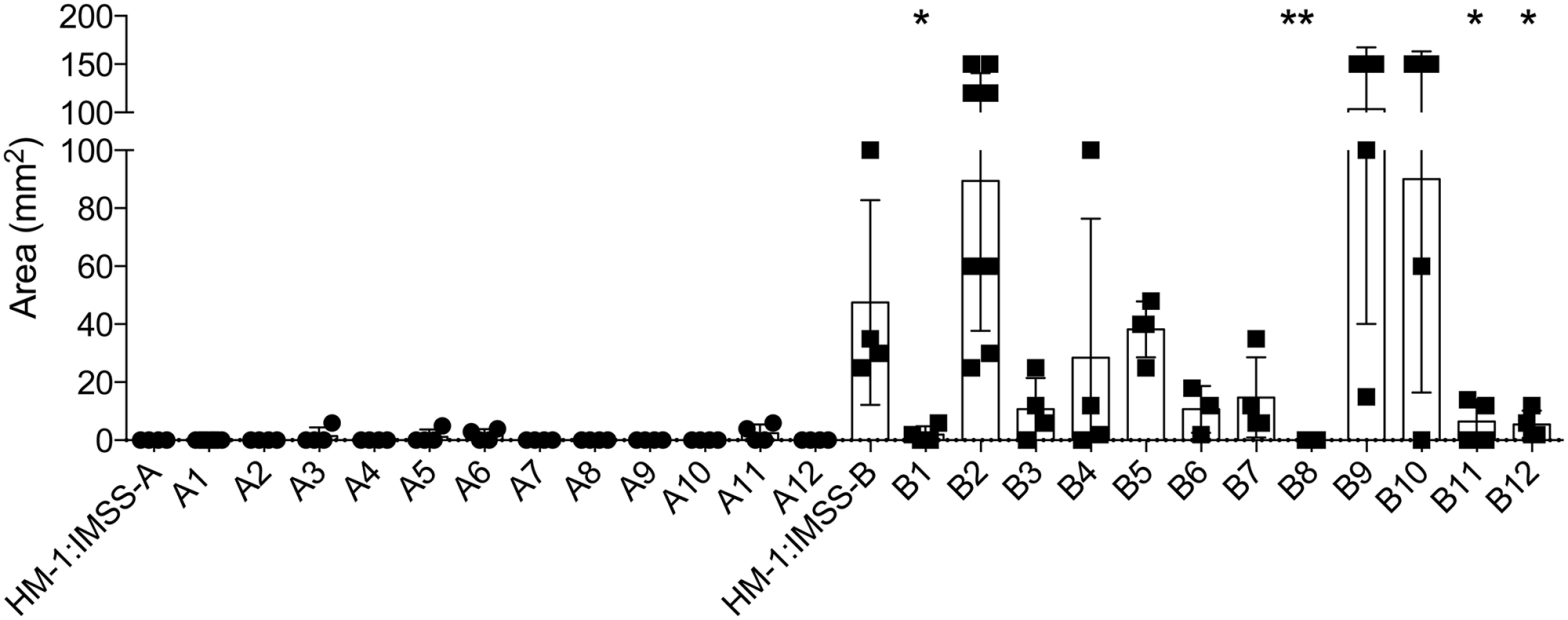
Analysis of ALA formation after infecting gerbils with various *E*. *histolytica* clones. Cell lines HM-1:IMSS-A and HM-1:IMSS-B were cloned by a limited dilution method, resulting in 12 clones derived from HM-1:IMSS-A (A1–A12) and 12 clones derived from HM-1:IMSS-B (B1–B12). Gerbils were infected with 1 × 10^6^ trophozoites of the different cell lines and clones. Seven days post infection, liver abscess formation was analysed and area of ALA (mm^2^) was determined. Significance (*p*-values) was established by the Mann-Whitney *U* test. *p*-values were calculated relative to control (HM-1:IMSS-A or HM-1:IMSS-B, respectively). *p = 0.0286, **p = 0.079. For detailed information see S1 Table.

To analyse whether the *E*. *histolytica* cell lines consisted of a mixture of different cell types with different pathogenic phenotypes, the cell lines were cloned by limited dilution method. This resulted in 12 clones derived from cell line HM-1:IMSS-A and 12 clones derived from cell line HM-1:IMSS-B. The ALAs-generating ability of the different clones was analysed using the gerbil model. The animals were sacrificed 7 days post infection, and ALA sizes were determined. The results clearly indicated that the HM-1:IMSS-A cell line consists of a homogenous cell population. Except a few cases of small ALA formation, the majority of animals infected with different A-clones showed no ALA formation (Fig 1). The results were more divergent for clones derived from the HM-1:IMSS-B cell line. Eight clones, B2–B7, B9 and B10, showed a pathogenic phenotype comparable with the original cell line HM-1:IMSS-B. However, although clones B1, B8, B11 and B12 were derived from the pathogenic cell line, their ability to induce abscess formation was significantly reduced. This was especially evident for clone B8 that did not induce any abscess formation 7 days post infection (Fig 1).

The non-pathogenic clones A1^np^ and B8^np^ and pathogenic clone B2^p^ have been continuously cultivated for more than 5 years, without any change of the respective phenotypes. The pathogenic phenotype of clone B2^p^ remained especially stable over the years without the need for animal passaging.

**Fig 2:**
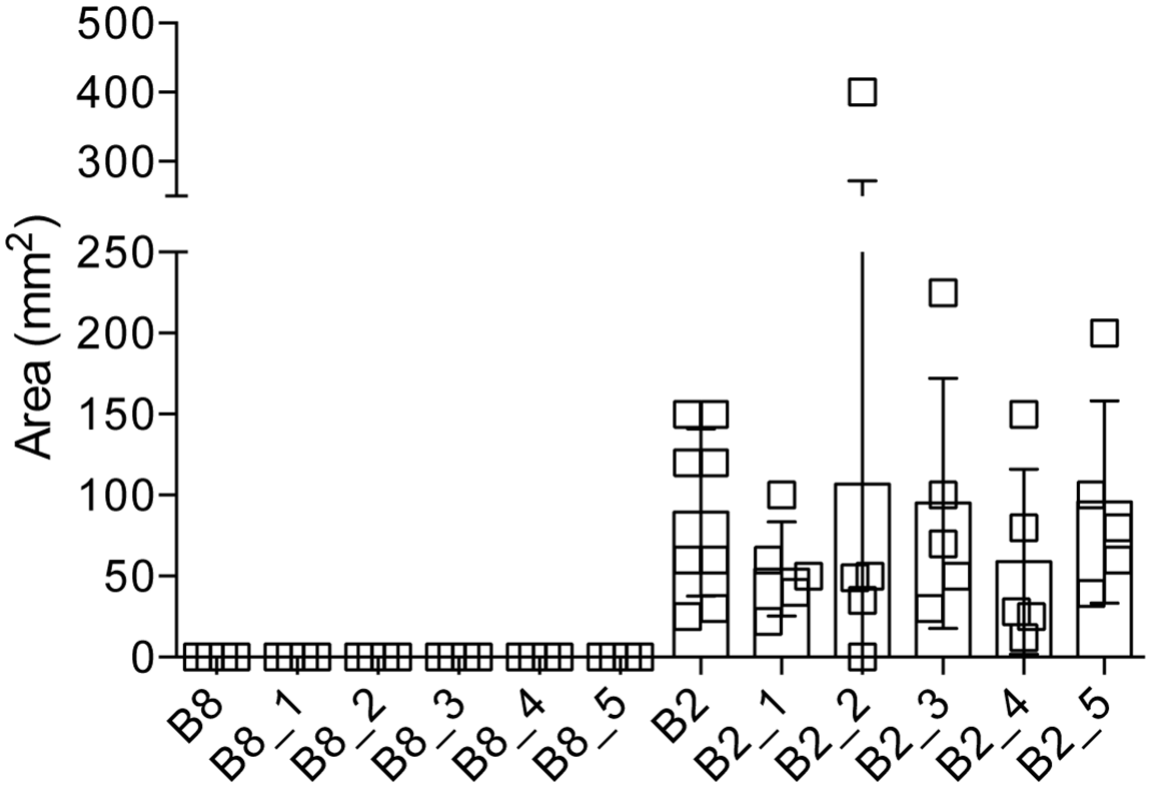
Analysis of ALA formation after infecting gerbils with various sub-clones derived from clone B8^np^ and clone B2^p^. Clones B8^np^ and B2^p^ were sub-cloned by a limited dilution method, resulting in five sub-clones each (B8_1–5 and B2_1–5, respectively). Gerbils were infected with 1 × 10^6^ trophozoites of the various sub-clones. Seven days post infection, liver abscess formation was analysed, and area of ALA (mm^2^) was determined. Significance (*p*-values) was established by the Mann-Whitney *U* test. *p*-values were calculated relative to control (clone B8^np^ or clone B2^p^, respectively). For detailed information see S1 Table.

To ensure that the observed phenotypes were indeed stable and uniform, the non-pathogenic clone B8^np^ and the highly pathogenic clone B2^p^ were sub-cloned. All sub-clones showed the same phenotype as the respective mother clone. All five sub-clones derived from the non-pathogenic clone B8^np^ were unable to induce ALAs, whereas all five sub-clones of the pathogenic clone B2^p^ produced large abscesses (Fig 2).

### Determination of cysteine peptidase activity

**Fig 3:**
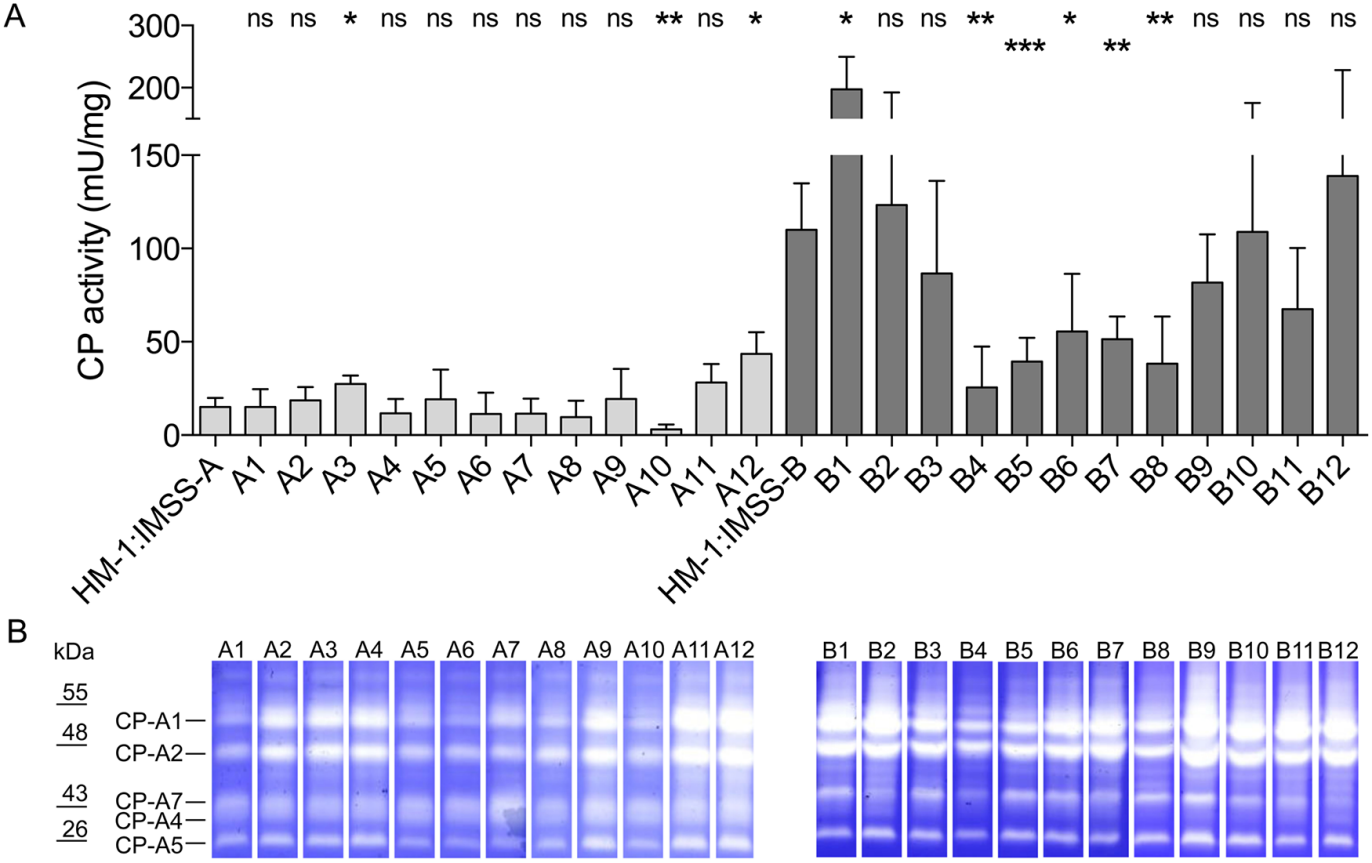
Determination of cysteine peptidase activity. (A) CP activities of cell line HM-1:IMSS-A, clones A1–A12, cell line HM-1:IMSS-B and clones B1–B12 were measured at least four times in duplicate. (B) Substrate gel electrophoresis of clones A1–A12 and clones B1–B12. Cell lysates were separated on SDS-PAGE co-polymerised with gelatine. To visualise CP activity of proteins, gels were stained with Coomassie blue and the images were inverted. Standards are indicated on the left (kDa). Significance (*p-*values) was established using an unpaired t test. *p*-values were calculated relative to control (HM-1:IMSS-A or HM-1:IMSS-B, respectively). *p < 0.05, **p < 0.005, ***p < 0.001, ns: not significant. For detailed information see S1 Table.

Recently, it was shown that CP activity of the pathogenic cell line HM-1:IMSS-B is approximately ten times greater (110 ± 25 mU/mg) than in the non-pathogenic cell line HM-1:IMSS-A (15 ± 5 mU/mg) [11]. A similar difference has been measured for the non-pathogenic clone A1^np^ (15 ± 10 mU/mg) and the pathogenic clone B2^p^ (123 ± 60 mU/mg) [3]. To investigate if the observed correlation between CP activity and pathogenicity is generally valid, the activities of all A- and B-clones were determined and correlated with ALA formation (Fig 3A). In general, the clones derived from HM-1:IMSS-B had a significantly higher CP activity (85 ± 50 mU/mg) than clones derived from HM-1:IMSS-A (18 ± 11; *p* < 0.0001). However, a direct correlation between the CP activity and ALA formation was not observed. This was obvious especially for clones B1 and B12. Although these clones have a high CP activity (198 ± 52 mU/mg and 139 ± 89 mU/mg, respectively), they only induce small ALAs (Figs 2 and 3A). CPs EhCP-A1, EhCP-A2, EhCP-A4, EhCP-A5 and EhCP-A7 can be visualised by substrate gel electrophoresis [3]. Here, the results from substrate gel experiments clearly indicated that different CP activities of the various A- and B-clones are not linked to the expression of a single peptidase (Fig 3B).

### Abscess formation over time

To identify the underlying mechanisms of different virulence phenotypes, three clones were selected for in-depth analyses. These were as follows: a non-pathogenic clone derived from HM-1:IMSS-A (clone A1^np^), pathogenic clone B2^p^ that induced the largest ALAs among the B-clones, and clone B8^np^ that completely lost its ability to produce ALAs. Both B-clones have been derived from HM-1:IMSS-B.

**Fig 4:**
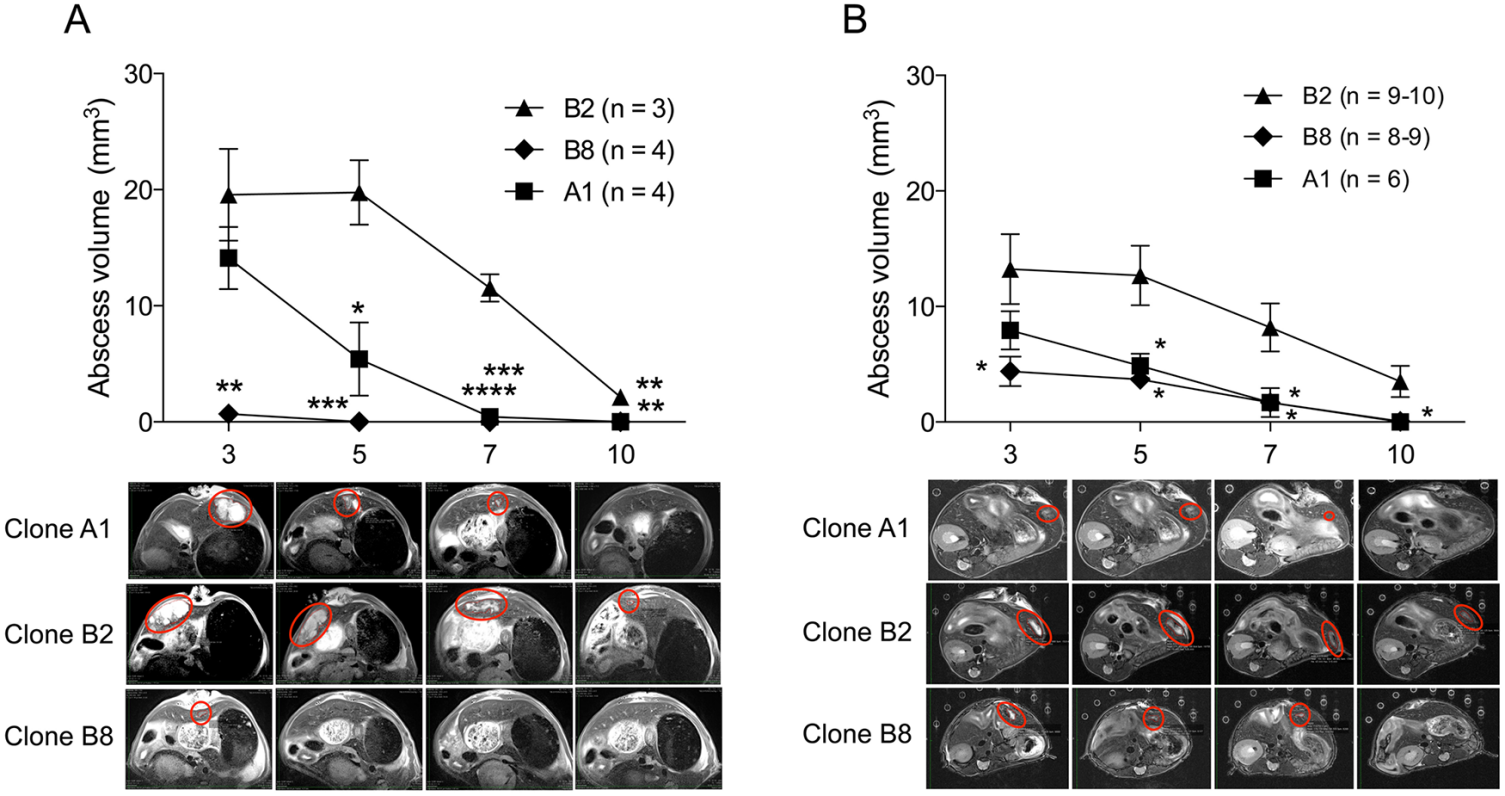
Analysis of the time course of ALA formation after infecting gerbils (A) and mice (B) with clones A1^np^, B2^p^ or B8^np^. Gerbils were infected with 1 × 10^6^ trophozoites, and mice were infected with 2.5 × 10^5^ trophozoites. Abscess sizes were determined on days 3, 5, 7 and 10 post infection using magnetic resonance imaging (MRI). Abscesses are shown in red circles. Significance was established using an unpaired t test. *p*-values were calculated relative to control (clone B2^p^). *p < 0.05, **p < 0.005, ***p < 0.001, ****p < 0.0001. For detailed information see S1 Table.

Magnetic resonance imaging (MRI) was employed to follow post-infection abscess formation over time in more detail. Infection time course was analysed over 10 days in gerbil and mouse ALA models using the three clones, A1^np^, B2^p^ and B8^np^ (Fig 4A and 4B). Our findings clearly confirmed the results of animal experiments described above (Fig 1). On day 7 post infection, no or very small ALAs were detected in both animal models with the two non-pathogenic clones A1^np^ and B8^np^, whereas unequivocal abscess formation was observed with clone B2^p^ (Fig 4A and 4B). Nevertheless, it became apparent that clone A1^np^ was also able to induce abscess formation initially, as lesions were detected on day 3 post infection. However, these ALAs were smaller compared with ALAs seen during clone B2^p^ infection and were more rapidly resolved. In contrast to clone A1^np^, the pathogenicity of clone B8^np^ was almost completely abolished. No ALAs were detected in gerbils infected with this clone, while, in mice, ALAs on day 3 post infection were significantly smaller compared with ALAs induced by clone B2^p^ (Fig 4A and 4B).

### Phenotypical characterisation of clones A1^np^, B2^p^ and B8^np^

Clones A1^np^, B2^p^ and B8^np^ were then phenotypically characterised. This included determination of size, growth rate, haemolytic activity, erythrophagocytosis and cytopathic activity.

Microscopic analyses indicated that trophozoite sizes of clone B2^p^ (818 ± 235 μm^2^) and clone B8^np^ (782 ± 193 μm^2^) differed significantly and were larger in comparison with cells from clone A1^np^ (591 ± 154 μM^2^) (Table 1).

**Table 1:** Phenotypical characterisation of clones A1^np^, B2^p^ and B8^np^.

Phenotype	Clone A1^np^	Clone B2^p^	Clone B8^np^
Abscess area (mm^2^)^a^	0	89 ± 52	0
Cysteine peptidase activity (mU/mg)^b^	15 ± 9.5	123 ± 69	38 ± 25
Size (μM^2^)^c^	591 ± 154	818 ± 235	782 ± 193
Growth rate (doubling time, h)^d^	12.02 ± 2.77	7.96 ± 1.63	9.49 ± 2.22
Haemolytic activity (%)^e^	6.85 ± 1.34	4.54 ± 0.63	0.79 ± 0.16
Erythrophagocytosis (OD_397nm_)^f^	1.1 ± 0.29	0.64 ± 0.17	1.59 ± 0.25
Cytopathic activity (percentage of monolyer disruption)^g^	53 ± 18	17 ± 29	0 ± 13
Motility (accumulated distance, μM/10 min)^h^	228±157	378±172	453±184

^a^Abscess area was analysed in the gerbil model 7 days post infection; n = 5–14; A1^np^/B2^p^ ****p < 0.0001; B2^p^/B8^np^ ***p = 0.0008

^b^n = 9–12; A1^np^/B2^p^ ****p < 0.0001; B2^p^/B8^np^; ***p = 0.0003; A1^np^/B8^np^ **p = 0.0093

^c^n = 80; A1^np^/B2^p^, A1^np^/B8^np^ ****p < 0.0001

^d^n = 10–15; A1^np^/B2^p^ ****p < 0.0001; A1^np^/B8^np^ *p = 0.0167

^e^n = 5–6; A1^np^/B2^p^, A1^np^/B8^np^, B2^p^/B8^np^ **p < 0.0043

^f^n = 8–12; A1^np^/B2^p^, B2^p^/B8^np^ ****p < 0.0001; A1^np^/B8^np^ *p = 0.011

^g^n = 18; A1^np^/B2^p^, A1^np^/B8^np^ ****p < 0.0001; B2^p^/B8^np^ *p = 0.0266

^h^n = 80; A1^np^/B2^p^, A1^np^/B8^np^ ****p < 0.0001, B2^p^/B8^np^ **p = 0.0013

Clone A1^np^ grew significantly slower in comparison with clone B2^p^ and clone B8^np^. The doubling time of clone A1^np^ was approximately 12 ± 2.7 h, whereas it was approximately 8 ± 1.6 h for clone B2^p^ and 9.5 ± 2.2 h for clone B8^np^ (Table 1).

With an accumulated distance of 228 (±156) μm/10 min the amoebae of clone A1^np^ move significantly slower in comparison to amoebae of clone B2^p^ and clone B8^np^ (376 ±172 μm/10 min (*p* < 0.0001) and 453 ±184 μm/10 min (*p* < 0.0001), respectively). While clone B8^np^ moved significantly faster than B2^p^ (*p* < 0.0013) (Table 1).

Clone A1^np^ and clone B2^p^ were able to lyse erythrocytes, but no haemolytic activity was detected for clone B8^np^. In addition, no correlation of haemolytic activity with pathogenicity was observed, since the non-pathogenic clone A1^np^ had the highest activity (Table 1). By contrast, clone B8^np^ displayed the highest erythrophagocytosis rate, followed by clone A1^np^ and clone B2^p^.

Cytopathic activity (percentage of monolayer disruption) was highest for clone A1^np^ followed by clone B2^p^. Clone B8^np^ was unable to disrupt a cell monolayer (Table 1).

### Transcriptome comparisons of clones A1^np^, B2^p^ and B8^np^

RNAseq experiments were performed to identify differences in gene expression profiles of clones A1^np^, B2^p^ and B8^np^. Comparison of the non-pathogenic clone A1^np^ and pathogenic clone B2^p^ revealed 76 differentially expressed genes (threshold ≥ 3-fold, *p*-value adjusted (padj) < 0.05). Some genes (46) were expressed more highly in clone A1^np^ and some (30) in clone B2^p^ (Tables 2 and 3, S2 Table). From the 46 genes with higher expression levels in clone A1^np^ 10 code for surface proteins (EHI_015290, EHI_082070, EHI_118130, EHI_169280, EHI_074080, EHI_075660 EHI_164900, EHI_039020, EHI_006170, EHI_086540) [13]. Amongst them are 2 members of the C2 domain protein family and 2 members of the Rab family. Since the analysis of the surface proteome referred to was performed with trophozoites of cell line A, it was not surprising that genes with higher expression levels in clone B2^p^ could not be identified as surface associated [13].

**Table 2:** Genes differentially more highly expressed in clone A1^np^ than in clone B2^p^ (threshold ≥ 3.0, padj ≤ 0.05).

SN	Gene ID	Expression level		Fold change	padj	Product
		Clone A1^np^	Clone B2^p^			
1	EHI_015290	5475.48	24.50	223.47	7.95E-52	C2 domain protein (EhC2-3)
2	EHI_042870	1097.95	7.36	149.12	1.15E-36	cell surface protease gp63 (EhMP8-2)
3	EHI_082070	732.36	7.75	94.48	9.72E-32	Rab family GTPase (EhRab7D)
4	EHI_059860	884.90	11.47	77.14	1.95E-32	C2 domain-containing protein (EhC2-5)
5	EHI_118130	1213.30	20.11	60.31	1.96E-26	C2 domain-containing protein (EhC2-2)
6	EHI_169280	287.26	5.67	50.61	2.47E-21	Rab family GTPase (EhRab7E)
7	EHI_074080	281.71	8.54	32.95	4.72E-14	hypothetical protein
8	EHI_187090	197.41	7.75	25.44	5.05E-14	Rab family GTPase (EhRab7G)
9	EHI_155060	20.53	2.76	7.42	0.0339	chaperone ClpB
10	EHI_005657	71.28	10.09	7.06	0.0012	hypothetical protein
11	EHI_151890	110.74	17.13	6.46	1.85E-05	EF-hand calcium-binding domain-containing protein
12	EHI_026360	1270.94	215.46	5.89	5.81E-05	phosphoserine aminotransferase (EhPSAT)
13	EHI_075660	297.80	50.93	5.84	1.40E-05	CAAX prenyl protease (EhCAAX)
14	EHI_097480	483.02	84.95	5.68	2.33E-05	hypothetical protein
15	EHI_075690	986.11	178.99	5.50	3.82E-07	hypothetical protein
16	EHI_137080	2264.50	437.89	5.17	2.32E-05	hypothetical protein
17	EHI_075640	1006.21	197.65	5.09	4.56E-06	protein phosphatase domain-containing protein
18	EHI_056490	2566.49	508.88	5.04	0.00011	20 kDa antigen
19	EHI_075700	181.71	36.77	4.94	0.00049	casein kinase II regulatory subunit family protein
20	EHI_164900	1176.80	246.96	4.76	8.44E-06	Rab family GTPase
21	EHI_114950	127.48	27.119	4.70	0.00046	hypothetical protein
22	EHI_039020	2024.47	448.98	4.50	2.75E-05	Actobindin
23	EHI_086690	3104.05	695.45	4.46	1.25E-05	hypothetical protein
24	EHI_175930	111.07	24.95	4.45	0.03065	hypothetical protein
25	EHI_086570	169.46	38.46	4.40	0.00069	hypothetical protein
26	EHI_028920	78.94	18.05	4.37	0.00092	heat shock protein
27	EHI_156560	76.06	17.79	4.27	0.00351	heat shock protein
28	EHI_126560	372.14	92.49	4.02	3.17E-06	AIG1 family protein
29	EHI_184500	257.72	64.63	3.98	6.78E-05	hypothetical protein
30	EHI_171750	140.38	35.85	3.91	0.00331	cysteine synthase 2
31	EHI_126550	225.41	57.93	3.89	0.00042	AIG1 family protein
32	EHI_034710	89.31	23.34	3.82	0.00139	heat shock protein
33	EHI_006170	979.62	257.71	3.80	0.01065	eukaryotic translation initiation factor 6
34	EHI_085470	227.82	62.12	3.66	0.0139	splicing factor3B subunit 1
35	EHI_148550	144.75	39.82	3.63	0.00041	protein tyrosine kinase domain-containing protein
36	EHI_042860	88.88	25.36	3.50	0.00353	heat shock protein
37	EHI_037550	134.83	38.51	3.50	0.00075	hypothetical protein
38	EHI_123830	380.95	109.06	3.49	0.00598	DNA mismatch repair protein Msh2
39	EHI_060340	625.57	179.63	3.48	0.00033	cysteine synthase A
40	EHI_022620	100.01	29.34	3.40	0.00090	heat shock protein
41	EHI_169470	12932.23	3978.28	3.25	0.00136	fructose-1,6-bisphosphate aldolase
42	EHI_086520	244.38	76.36	3.20	0.00127	3' exoribonuclease family protein
43	EHI_177570	421.06	134.05	3.14	0.00123	RNA 3'-terminal phosphate cyclase
44	EHI_116830	1096.68	353.71	3.10	3.47E-05	d-phosphoglycerate dehydrogenase
45	EHI_075710	187.29	60.76	3.08	0.03569	hypothetical protein
46	EHI_086540	205.49	66.68	3.08	0.00933	replication factor C subunit 4

Padj: Adjusted *p*-value, SN: Serial number

**Table 3:** Genes differentially more highly expressed in clone B2^p^ than in clone A1^np^ (threshold ≥ 3.0, padj ≤ 0.05).

SN	Gene ID	Expression level		Fold change	padj	Product
		Clone B2^p^	Clone A1^np^			
1	EHI_127670	2314.73	12.01	192.59	0.00029	hypothetical protein
2	EHI_144490	3579.49	19.75	181.19	8.61E-45	hypothetical protein
3	EHI_169670	2551.64	27.28	93.5	1.59E-08	hypothetical protein
4	EHI_050490	25.89	0.46	55.61	6.78E-05	hypothetical protein
5	EHI_062080	123.01	5.33	23.04	0.00073	hypothetical protein
6	EHI_178610	1265.3	81.56	15.51	9.25E-07	tyrosine kinase
7	EHI_013240	1500.57	104.17	14.4	2.36E-27	hypothetical protein
8	EHI_076370	29.83	2.31	12.86	0.00073	hypothetical protein
9	EHI_121820	937.251	73.14	12.81	6.82E-27	hypothetical protein
10	EHI_111330	70.77	7.35	9.62	1.38E-06	hypothetical protein
11	EHI_192510	110.82	12.8	8.65	0.04327	hypothetical protein
12	EHI_017760	343.35	43.45	7.9	2.97E-05	tyrosine kinase
13	EHI_165190	84.71	12.04	7.03	2.46E-05	hypothetical protein
14	EHI_014170	4757.56	803.79	5.91	9.48E-16	hypothetical protein
15	EHI_101630	316.38	68.53	4.61	0.03397	hypothetical protein
16	EHI_144610	2146.29	505.62	4.24	0.00275	methionine gamma-lyase
17	EHI_029360	308.73	74.75	4.13	0.02649	hypothetical protein
18	EHI_057550	1835.39	465.22	3.94	0.00063	methionine gamma-lyase
19	EHI_029380	49.86	12.94	3.85	0.02703	Thioredoxin
20	EHI_176590	226.19	60.29	3.75	0.00115	AIG1 family protein
21	EHI_150770	752.48	205.27	3.66	0.00024	heat shock protein 70
22	EHI_197980	176.61	49.70	3.55	4.18E-05	Myb family DNA-binding protein
23	EHI_104570	2203.52	649.79	3.39	0.00020	ubiquitin ligase
24	EHI_063550	423.11	126.66	3.34	4.60E-06	Myb-like DNA-binding domain-containing protein
25	EHI_062970	128.26	38.68	3.31	0.00524	hypothetical protein
26	EHI_087000	502.04	153.12	3.27	0.01391	hypothetical protein
27	EHI_033700	560.27	172.86	3.24	0.00033	RecF/RecN/SMC domain-containing protein
28	EHI_063580	167.63	52.23	3.20	0.00160	hypothetical protein
29	EHI_131490	226.62	73.84	3.06	0.00049	leucine-rich repeat protein. BspA family
30	EHI_020250	635.65	207.17	3.06	0.04815	lecithin:cholesterol acyltransferase domain-containing protein

Padj: Adjusted *p*-value, SN: Serial number

The greatest differences, with expression fold changes ~20–200, concerned genes encoding three C2 domain proteins, three Rab family GTPases, cell surface protease gp63 and one hypothetical protein (EHI_074080), whose expression was higher in clone A1^np^; and five genes encoding hypothetical proteins EHI_127670, EHI_144490, EHI_169670, EHI_050490 and EHI_062080, with higher expression in clone B2^p^. The majority of the identified genes showed 3-4-fold differential expression (Tables 2 and 3). The detected differential expression was verified by quantitative real-time PCR (qPCR) for 26 genes. Results of next generation sequencing were confirmed for all the analysed genes, with the exception of EHI_056490 (threshold ≥ 2 fold, Table 4).

**Table 4:** mRNASeq and quantitative real-time PCR data for genes whose expression was at least ≥ 3-fold higher in clone A1^np^ than in clone B2^p^.

			ddCT^a^	
	Gene ID	mRNASeq (fold change)	Clone A1^np^	Clone B2^p^ (calibrator)
EhC2-3 (C2 domain-containing protein)	EHI_015290	223.47	254.6 ± 229.6**	1
EhMP8-2 (cell surface protease gp63)	EHI_042870	149.12	134.7 ± 54.93**	1
EhRab7D (Rab family GTPase)	EHI_082070	94.48	22.5 ± 15.64***	1
EhC2-5 (C2 domain-containing protein)	EHI_059860	77.14	490.2 ± 88.81*	1
EhC2-2 (C2 domain-containing protein)	EHI_118130	60.31	66.2 ± 3.3*	1
EhRab7E (Rab family GTPase)	EHI_169280	50.61	42.1 ± 28.31****	1
Hypothetical protein	EHI_074080	32.95	229.3 ± 99.95***	1
EhRab7G (Rab family GTPase)	EHI_187090	25.44	28.2 ± 24.52***	1
Hypothetical protein	EHI_005657	7.06	2.87 ± 0.4****	1
EhCAAX (CAAX prenyl protease)	EHI_075660	5.84	2.5 ± 0.83 **	1
Hypothetical protein	EHI_075690	5.50	5.6 ± 2.8***	1
Protein phosphatase domain-containing protein	EHI_075640	5.09	2.8 ± 0.3*	1
20 kDa antigen	EHI_056490	5.04	1.9 ± 0.14*	1
Casein kinase II regulatory subunit family protein	EHI_075700	4.94	2.7 ± 1*	1
Hypothetical protein	EHI_086690	4.46	3.3 ± 0.13*	1
Replication factor C subunit 4	EHI_086540	3.08	2.5 ± 0.23*	1
			**Clone A1** ^**np**^ **(calibrator)**	**B2** ^**p**^
Hypothetical protein	EHI_127670	192.59	1	13.5 ± 5*
Hypothetical protein	EHI_144490	181.19	1	38 ± 6.4*
EhC2-DIL1 (hypothetical protein)	EHI_169670	93.5	1	58.5 ± 58****
Tyrosine kinase	EHI_178610	15.51	1	14.8 ± 9**
Hypothetical protein	EHI_013240	14.4	1	6.9 ± 0.07*
Hypothetical protein	EHI_121820	12.81	1	6 ± 1.3**
Hypothetical protein	EHI_111330	9.62	1	9.4 ± 1.2**
Hypothetical protein	EHI_165190	7.03	1	4.8 ± 2.6**
EhC2-DIL2 (hypothetical protein)	EHI_014170	5.91	1	57.4 ± 38.1****
Methionine gamma-lyase	EHI_144610	4.24	1	2.6 ± 0.4*

^a^Relative differences in gene expression; ehactin was used as a normalizer.

*p ≤ 0.05,

**p ≤ 0.005,

***p ≤ 0.001,

****p ≤ 0.0001.

Most of the identified genes encode proteins with unknown function. Some of these hypothetical proteins contain functional domains, e.g., C2 domains, phosphatase domains, tyrosine kinase domains, RecF/RecN/SMC domains and lecithin:cholesterol acyltransferase domains. Proteins with known functions up-regulated in clone A1^np^, when compared with clone B2^p^, mainly included Rab family proteins, peptidases and heat shock proteins. Putative function could be assigned to only 6/30 genes identified as more highly expressed in clone B2^p^ in comparison with clone A1^np^ (e.g., tyrosine kinase, methionine gamma-lyase, thioredoxin) (Tables 2 and 3).

Comparisons of the two B-clones, B2^p^ and B8^np^, revealed only 19 differentially expressed genes. Twelve genes were expressed at higher levels in clone B8^np^ in comparison with clone B2^p^, and seven genes were expressed more highly in B2^p^ in comparison with B8^np^ (Tables 5 and 6, S3 Table). The corresponding proteins assigned to EHI_039020 and EHI_088020 were found to be part of the surface proteome of *E*. *histolytica* [13]. Fold change ≥ 10 was detected for only three genes. These genes encoded two hypothetical proteins and a leucine-rich repeat-containing protein. All these genes were more highly expressed in B2^p^ in comparison with B8^np^. As observed in the A1^np^/B2^p^ comparison, the majority of the identified genes showed 3-4-fold differential expression (Tables 5 and 6). The majority (11/19) of the genes encoded hypothetical proteins. The remaining genes were annotated as galactose-inhibitable lectin 35 kDa subunit, phosphoserine aminotransferase, actobindin, alcohol dehydrogenase, AIG1 family protein and methionine gamma-lyase. However, only galactose-inhibitable lectin 35 kDa subunit and methionine gamma-lyase have been biochemically characterised in *E*. *histolytica* [14–17].

**Table 5:** Genes differentially more highly expressed in clone B8^np^ than in clone B2^p^ (threshold ≥ 3.0, padj ≤ 0.05).

SN	Gene ID	Expression level		Fold change	padj	Product
		Clone B8^np^	Clone B2^p^			
1	EHI_062960	64.90	7.85	8.26	0.00806	hypothetical protein
2	EHI_183400	37.64	5.66	6.64	0.02770	galactose-inhibitable lectin 35 kDa subunit
3	EHI_048140	771.02	160.91	4.79	0.00114	hypothetical protein
4	EHI_058920	1548.15	360.65	4.29	7.462E-08	hypothetical protein
5	EHI_026360	137.56	33.05	4.16	0.00114	phosphoserine aminotransferase
6	EHI_037160	102.17	26.67	3.83	0.01207	hypothetical protein
7	EHI_039020	356.95	101.96	3.50	0.03229	Actobindin
8	EHI_088020	821.37	234.45	3.50	0.28544	alcohol dehydrogenase
9	EHI_151930	521.04	156.15	3.33	0.00017	hypothetical protein
10	EHI_160670	252.40	76.60	3.29	0.00514	alcohol dehydrogenase 3
11	EHI_180390	216.17	69.98	3.09	0.00611	AIG1 family protein
12	EHI_056490	290.45	98.02	2.96	0.00514	20 kDa antigen

Padj: Adjusted *p*-value, SN: Serial number

**Table 6:** Genes differentially more highly expressed in clone B2^p^ than in clone B8^np^ (threshold ≥ 3.0, padj ≤ 0.05).

SN	Gene ID	Expression level		Fold change	padj	Product
		Clone B2^p^	Clone B8^p^			
1	EHI_127670	806.03	17.69657648	45.54	0.00017	hypothetical protein
2	EHI_073680	149.05	19.46774451	14.31	8.914E-17	leucine-rich repeat-containing protein
3	EHI_144490	1086.69	94.76770315	11.46	5.624E-22	hypothetical protein
4	EHI_062080	6.71	40.72199698	6.07	0.02179	hypothetical protein
5	EHI_191730	96.40	27.58393181	3.49	0.01942	hypothetical protein
6	EHI_144610	661.91	201.7370164	3.28	0.00017	methionine gamma-lyase
7	EHI_057550	556.94	175.7481139	3.16	0.00046	methionine gamma-lyase

Padj: Adjusted *p*-value, SN: Serial number

Interestingly, only six genes in the two non-pathogenic clones A1^np^ and B8^np^ were similarly regulated vs. pathogenic clone B2^p^. Genes EHI_026360, EHI_039020 and EHI_056490 were up-regulated, and genes EHI_127670, EHI_144490 and EHI_144610 were down-regulated, in clones A1^np^ and B8^np^ in relation to clone B2^p^. This suggests different mechanisms accounting for the inability of clones A1^np^ and B8^np^ to induce ALAs.

### Characteristics of the proteins encoded by genes found to be differentially expressed between clone A1^np^ and clone B2^p^ and/or clone B8^np^ and clone B2^p^

In total 89 genes were found to be differentially expressed between clone A1^np^ and clone B2^p^ and/or between clone B8^np^ and clone B2^p^. In a previous study, the transcriptomes of the non-clonal cell lines A and B were compared using a microarray approach [12]. Here, in total 31 genes were differentially expressed (threshold ≥3-fold). Of the 12 genes with higher expression levels in cell line B in comparison to cell line A, 7 genes had also higher expression levels in clone B2^p^ in comparison to clone A1^np^. Out of the 19 genes with higher expression levels in cell line A in comparison to cell line B, 11 genes showed also higher expression in clone A1^np^ in comparison to clone B2^p^ (S4 Table). Only 8 of the identified 89 genes encoded for proteins containing a signal peptide and 15 genes encoded for proteins containing between 1–7 transmembrane domains (S4 Table). In a previous study in which the surface proteome of cell line A was analysed, 693 putative surface-associated proteins were identified [13]. Out of them 11 showed differential expression between the different clones (S4 Table).

From the 89 identified genes, 32 encode hypothetical proteins, where no homology to other proteins or protein domains could be identified.

Furthermore, 3 genes encode for proteins of the C2 superfamily, 4 genes encode for members of the small GTPase superfamily, 10 genes encode for heat shock proteins, 6 genes encode for AIG1 family proteins, 3 genes encode for kinases 2 genes encode for cysteine synthases and 2 genes encode for proteases. Additional 18 genes encode for proteins with other known functions (S4 Table).

### Transfectants overexpressing a selection of the differentially expressed genes and gene involvement in ALA formation

**Fig 5:**
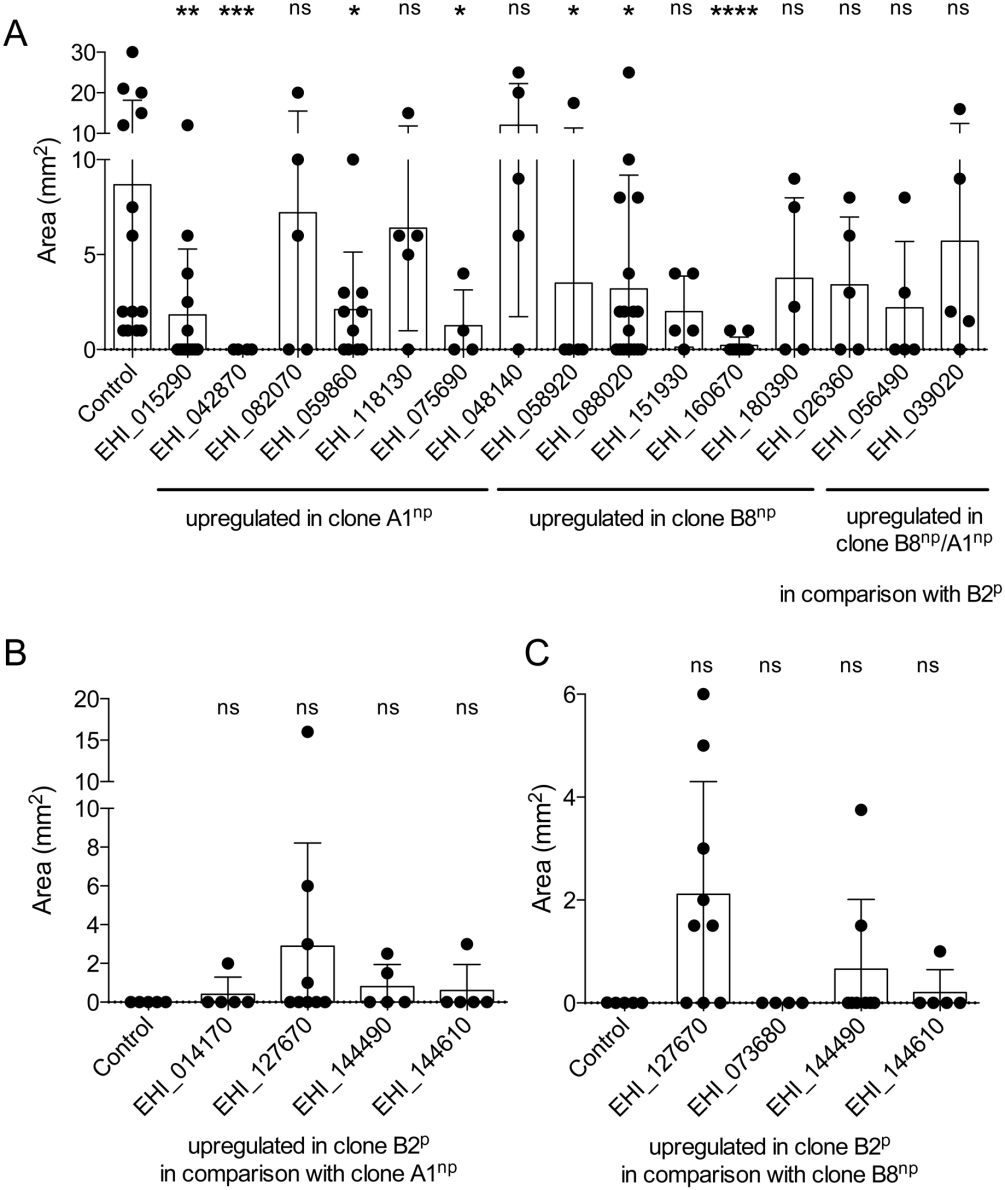
Analysis of ALA formation after infecting gerbils with various *E*. *histolytica* transfectants. B2^p^ (A), A1^np^ (B) and B8^np^ transfectants (C) overexpressing various genes differentially expressed in clones A1^np^ and B2^p^ were used to intrahepatically infect mice. Seven days post infection, ALA formation was determined [area of ALA (mm^2^)]. Significance (*p*-values) was established by the Mann-Whitney *U* test. *p*-values were calculated relative to control (pNC transfectants of clone B2^p^ (A), A1^np^ (B) or B8^np^ (c), respectively). *p < 0.05, **p < 0.005, ***p < 0.001, **** *p* < 0.0001, ns: not significant. For detailed information see S1 Table.

To investigate whether the differentially expressed genes play a role in ALA formation, their respective overexpressing transfectants were generated. For genes that were more highly expressed in clone A1^np^ in comparison with clone B2^*p*^, 13 overexpressing transfectants of clone B2^p^ were generated. These included eight genes that displayed the highest differential expression (>20-fold) and three genes that were also up-regulated in clone B8^np^ (EHI_026360, EHI_056490, EHI_039020) (Tables 2 and 5). We were unable to generate transfectants overexpressing genes EHI_169280 (*ehrab7e*), EHI_074080, EHI_187090 (*ehrab7g*) and EHI_075660 (*ehcaax*). For all other genes, a relative 2.4–235-fold overexpression was obtained (S5 Table). The pathogenic phenotype of the majority of B2^p^ transfectants overexpressing genes that were more highly expressed in the non-pathogenic clone A1^np^ vs. pathogenic clone B2^p^ was unaffected. These also included transfectants that overexpressed three genes regulated in the same manner in clones A1^np^ and B8^np^. All these B2^p^ transfectants induced ALA formation in mice. Four genes were identified whose overexpression had a dramatic impact on ALA formation. When EHI_015290 (*ehc2-3*), EHI_059860 (*ehc2-5*), EHI_042870 (*ehmp8-2*) or EHI_075690 were overexpressed in clone B2^p^, these clones lost their pathogenic phenotype and produced significantly smaller ALAs than the respective controls (Fig 5A).

B2^p^ transfectants were generated for 9/12 genes that were expressed at higher levels in clone B8^np^ in comparison with clone B2^p^. They showed 4–300-fold increased expression in comparison with the control (S5 Table). Overexpression of genes EHI_058920, EHI_088020 and EHI_160670 significantly reduced pathogenicity of clone B2^p^ (Fig 5A). *In silico* analyses indicated that nucleotide sequences of EHI_088020 and EHI_160670 were identical and that the genes encode an alcohol dehydrogenase. However, the first 480 nucleotides of the EHI_088020 coding region were missing from the EHI_160670 sequence. This may be because the *E*. *histolytica* genome is not yet fully annotated (AmoebaDB, http://amoebadb.org/amoeba/). Regardless, increased expression of the full-length or truncated gene impacted abscess formation (Fig 5A).

For genes expressed more highly in clone B2^p^ in comparison with clone A1^np^, five gene-overexpressing clone A1^np^ transfectants were generated, including three genes with an initially detected >90-fold differential expression. Overexpression of EHI_169670 was unsuccessful; however, for all other genes a relative 4–490-fold expression was obtained (S6 Table). Overexpression of these genes did not significantly affect ALA formation. However, strikingly, 4/9 mice infected with EHI_127670-overexpressing A1^np^ transfectant produced large ALAs (Fig 5B).

Seven genes were more highly expressed in clone B8^np^ in comparison with clone B2^p^. Two, EHI_144610 and EHI_057550, were identical and encode methionine gamma-lyase. Four B8^np^ transfectants were generated, and all of them showed 3–70-fold overexpression in comparison with the control (S7 Table). Similarly to A1^np^ transfectants, no significant influence on ALA formation was observed. Interestingly, large ALAs were detected 7 days post infection in 6/9 mice infected with B8^np^ transfectant overexpressing EHI_127670 (p = 0.0589), as was observed for infections with A1^np^-EHI_127670 transfectants (p = 0.292) (Fig 5B and 5C).

## Discussion

Recent studies aiming to identify the differences between the non-pathogenic isolate HM-1:IMSS_A and the closely related but pathogenic isolate HM-1:IMSS_B using proteomic and transcriptomic analyses revealed a surprisingly small overlap between the two approaches [11, 12]. To eliminate the potential experimental limitations associated with cell population heterogeneity, we cloned both mother lines resulting in 12 clones derived from cell line HM-1:IMSS_A (A1–A12) and 12 clones derived from cell line HM-1:IMSS_B (B1–B12). As expected, all clones derived from cell line HM-1:IMSS_A were unable to form ALAs. Surprisingly, the situation was more complex for clones derived from the HM-1:IMSS_B cell line. Here, only 8/12 clones analysed displayed the pathogenic phenotype of the original cell line. To understand the phenotypically different outcomes, three clones were selected for in-depth analyses. These were pathogenic clone B2^p^ and non-pathogenic clones A1^np^ and B8^np^.

Interestingly, and in contrast to other pathogenic isolates, the pathogenicity of clone B2^p^ remained stable for years without animal passage. Usually, trophozoites become less virulent after long-term culture and pathogenicity retainment requires regular animal passages or at least the addition of cholesterol to the culture medium [18–22].

Although the mouse model for ALA, has some limitations regarding the artificial route of infection, it is well established [23–25] and allows in this study to discriminate between pathogenic and less pathogenic clones. Accordingly, the high reproducibility of the differences in the recovery time of the liver between „non-pathogenic”and „pathogenic”*E*. *histolytica* clones opens a stable time frame that enables studying pathogenicity factors involved in liver pathology on a significant level. However, no conclusion can be drawn concerning the process of invasion into the intestinal mucosa, induction of amoebic colitis and immune evasion [26, 27].

CPs have been described as major pathogenicity factors of *E*. *histolytica*. In several studies, a direct correlation between CP activity and ALA formation was observed [19, 28–31]. In addition, ALA formation can be inhibited by specific cysteine peptidase inhibitors, and overexpression and silencing of individual *E*. *histolytica cp* genes can alter the ALAs-inducing ability of amoebae [3, 6, 32–36]. Furthermore, several studies indicate that especially EhCP-A5 is involved in the invasion process into the intestinal mucosa [37–39]. In this context, it was shown that EhCP-A5 triggers the production and release of human matrix metalloproteinases (MMPs) through inflammatory cytokine induction. Moreover it cleaves pro-MMP-3 and converts it into active MMP-3. Together, these processes are involved in the ECM remodelling required for tissue invasion [38]. Furthermore, it was shown that EhCP-A5 abrogates the MUC2 protective function by cleavage of the MUC2 C-terminus and that EhCP-A5 also plays an role in contact-dependent mucin hypersecretion during intestinal amebiasis [39]. Correlation between CP activity and ALA formation was described for the non-pathogenic cell line HM-1:IMSS_A (CP activity, ~15 mU/mg) and the pathogenic cell line HM-1:IMSS_B (CP activity, ~110 mU/mg), as well as clones B2^p^ (CP activity, ~120 mU/mg) and A1^np^ (CP activity, ~15 mU/mg) [3, 11]. However, determination of CP activities, especially in B-clones, did not support these observations. Prominent examples of this discrepancy are clones B1 and B12, which are non-pathogenic but have the highest CP activity of all the clones analysed (~150–200 mU/mg). Substrate gel electrophoresis experiments indicated that these different levels of CP activity result from altered abundances of all major CPs rather than from changes of individual CPs. Such lack of correlation between CP activity levels and ALA formation was reported in only one other publication, where Montfort and colleagues also used pathogenic and non-pathogenic cultures of *E*. *histolytica* isolate HM-1:IMSS [21]. However, the missing correlation between CP activity and ALA formation is not necessarily inconsistent with previous results. Therefore, it can’t be excluded that a high CP activity alone is not sufficient to induce ALAs. Other factors may be involved that, in combination with the CPs, lead to ALA formation. Furthermore, it was recently shown, that the different CPs have different impact on ALA formation. It was shown that overexpression of *ehcp-a5*, one of the major expressed *ehcps*, but also of the very low expressed *ehcps*, *ehcp-b8*, *-b9*, and *-c13* restored the pathogenic phenotype of the non-pathogenic clone A1^np^, whereas overexpression of various other peptidase genes including the major expressed *ehcp-a1* and *ehcp-a2* had no effect on pathogenicity [3]. In addition, in the present study the expression level of the genes under culture conditions were compared between the different clones. However it was recently shown, that the expression level of some *ehcp* genes (*ehcp-a3*, *-a4*, *-a5*, *-a6*, *-a10*, *-b8*, *-b9*, *and -c13*) increased during ALA formation [3]. Therefore, it can be speculated, that the non-pathogenic amoebae lost their ability to regulate the expression of the peptidases under altered environmental conditions.

In addition to CP activity, other frequently used *in vitro* pathogenicity markers are erythrophagocytosis, haemolytic activity and cytopathic activity. In contrast with most reports that described a correlation between erythrophagocytosis and ALA formation [22, 40–43], Monfort and colleagues and Tsutsumi and colleagues, as well as this study, did not confirm this correlation [21, 44]. In the present study, non-pathogenic clone B8^np^ showed the highest erythrophagocytosis rate, followed by clones A1^np^ and B2^p^. Recently, a mechanism described as amoebic trogocytosis was identified, where amoebae ingest “bites” of host cells [45, 46]. Interestingly, only living cells were ingested by trogocytosis, whereas dead cells were phagocytosed in total. So far, we have no hint if amoebic trogocytosis correlates with the different ability of clone A1^np^, B2^p^ and B8^np^ to from ALAs. However, none of the molecules with known function in amoebic trogocytosis of erythrocytes were found to be differentially expressed in either of the three clones [45].

Haemolytic activity has also been reported as related to pathogenicity [22, 47]. However, similarly to what was described decades ago by Keller and colleagues [48], we did not observe any correlation of this trait with virulence. We showed that non-pathogenic clone B8^np^ was unable to lyse erythrocytes, while non-pathogenic clone A1^np^ had a higher haemolytic activity than pathogenic clone B2^p^. Furthermore, it was recently shown that haemolytic activity of the non-pathogenic cell line HM-1:IMSS_A is significantly higher than that of the pathogenic cell line HM-1:IMSS_B [11].

It is indisputable that cytopathogenicity is an important feature for *E*. *histolytica* pathogenicity [49, 50]. However, to the best of our knowledge, no study has correlated cytopathogenicity with virulence. A comparison of the abilities of clones A1^np^, B2^p^, and B8^np^ to disrupt a CHO cell monolayer showed that clone A1^np^ had the highest cytopathic activity, whereas clone B8^np^ had no cytopathic activity. Therefore, even when cytopathic activity was taken into consideration, no correlation with ALA formation could be found.

The non-pathogenic clone A1^np^ exhibits a higher haemolytic and cytopathic activity in contrast to clone B8^np^, while the motility of clone B8^np^ is higher compared to clone A1^np^. From these phenotypical observations we speculate that pathogenicity of the parasite might require i) the ability to destroy and ii) phagocytose host cells and iii) to exhibit a certain motility, parameters which we find to be combined in clone B2^p^. Finally we conclude that the ability of *E*. *histolytica* to destroy liver tissue involves complex processes, from both, the parasite and the host side [24].

The genome of *E*. *histolytica* comprises ~8400 genes. Matching the transcriptomes of clones A1^np^, B2^p^ and B8^np^ to one another revealed that only a minority of genes were differentially transcribed. Comparing the transcriptomes of non-pathogenic clone A1^np^ and pathogenic clone B2^p^ revealed 46 genes that were more highly expressed in clone A1^np^ and 30 genes that were more highly expressed in clone B2^p^ (≥3-fold). The expression of 60% [18/31] of the genes that were differentially expressed in mother cell lines HM-1:IMSS_A and HM-1:IMSS_B [12] was also significantly different for clones A1^np^ and B2^p^. These included genes encoding Rab family GTPases (EhRab7D, EHI_082070; EhRab7E, EHI_169280; EhRab 7G, EHI_187090), C2 domain-containing protein (EhC2-2, EHI_118130) and cell surface protease gp63 (EhMP8-2, EHI_042870). Comparison of the pathogenic clone B2^p^ with the non-pathogenic clone B8^np^, both derived from pathogenic cell line HM-1:IMSS_B, revealed only 19 differentially expressed genes (≥3-fold). Of these, 12 were more highly expressed in clone B8^np^ and seven were more highly expressed in clone B2^p^. Only six genes were regulated in the same manner in the two non-pathogenic clones A1^np^ and B8^np^ (EHI_026360, EHI_056490, EHI_039020, EHI_127670, EHI_144490 and EHI_144610).

Since the identified differentially expressed genes encode proteins of divergent function or hypothetical proteins, it is speculative whether these genes correlate with pathogenic amoeba phenotype. To clarify this issue, we generated overexpression transfectants for a set of candidate genes. Six genes up-regulated in clone A1^np^, six genes up-regulated in clone B8^np^ and three genes up-regulated in clones A1^np^ and B8^np^, in comparison with B2^p^, were overexpressed in B2^p^. Significant reduction in abscess size in comparison with the control was determined for 7/15 B2^p^ transfectants. The respective overexpressed genes encoded two C2 domain proteins (EhC2-3, EHI_15290; EhC2-5, EHI_05980), cell surface protease gp63 (EhMP8-2, EHI_042870), two alcohol dehydrogenases (EHI_088020, EHI_160670) and two hypothetical proteins (EHI_075690, EHI_058920).

Within the *E*. *histolytica* genome, four genes (EHI_069320, EHI_118130, EHI_015290, EHI_059860) were identified as encoding C2 domain proteins that have 60–75% identity. Three of these were more highly expressed in clone A1^np^ compared with clone B2^p^, and for all three the respective B2^p^ transfectants were generated. Overexpression of EHI_015290 (EhC2-3) and EHI_059860 (EhC2-5) significantly reduced pathogenicity of clone B2^p^. In general, C2 domains are involved in targeting proteins to cell membranes. Thus far, only one (EHI_069320, EhC2-1) of the C2 domain proteins has been characterised. It mediates anchoring of the transcription factor URE3-BP to the amoebic plasma membrane [51].

Two cell surface protease gp63 homologues (EHI_200230, EhMP8-1; EHI_042870, EhMP8-2) are encoded in the *E*. *histolytica* genome. EhMP8-2 was more highly expressed in clone A1^np^ in comparison with clone B2^p^. This was also true for the two cell lines HM-1:IMSS_A and HM-1:IMSS_B [12]. However, no differential expression was observed for EhMP8-1. Both metalloproteases belong to the M8 family zinc metalloproteases with homology to leishmanolysin, a protein essential for virulence of *Leishmania* [52, 53]. They contain a zinc-binding HEXXH catalytic site motif and a putative transmembrane domain, and have 34% identity with each other. EhMP8-1 is localised on the trophozoite surface, and further characterisation revealed involvement in adherence, mobility, cytopathogenic activity and phagocytosis [54]. There is no indication that EhMP8-2 exhibits similar functions, since its expression levels in different clones did not correlate with cytopathogenic activity and phagocytosis.

The expression of EHI_075690 was five times higher in A1^np^ than in B2^p^. The gene EHI_075690 encodes a 218-amino acid hypothetical protein. *In silico* analysis revealed that the protein consists of four transmembrane domains with homology to tetraspanin family proteins. Until now, six tetraspanins were identified in the *E*. *histolytica* genome; however, their function is mostly unknown [55]. In general, tetraspanins are known to be involved in cell proliferation, adhesion, signalling and migration [56]. Recently, it was shown that the tetraspanin TvTSP8 of *Trichomonas vaginalis* is involved in parasite-parasite communication [57].

EHI_058920 was more highly expressed in clone B8^np^ in comparison with clone B2^p^, and overexpression reduced the pathogenicity of clone B2^p^. The gene encodes a protein of 316 amino acids. No homologues in other organisms and no conserved domains were identified within the protein.

EHI_088020 and EHI_160670, both more highly expressed in clone B8^np^ in comparison with clone B2^p^, encode alcohol dehydrogenases with the highest homology to Fe-dependent dehydrogenases of Gram-negative obligatorily anaerobic prokaryotes. Therefore, it may be assumed that they were incorporated into the amoebal genome by lateral gene transfer. As mentioned above, *in silico* analyses indicated that amino acid sequences of proteins encoded by EHI_088020 and EHI_160670 are identical; however, the first 160 amino acids of EHI_088020 are missing from the EHI_160670 sequence. Since EHI_160670 is located at the 5′-end of the published contig DS571485, this ‘deletion’ may be explained by a not-fully annotated status of the *E*. *histolytica* genome (AmoebaDB, http://amoebadb.org/amoeba/). However, we were unable to identify the sequence upstream of EHI_160670. Nevertheless, ectopic expression of the full-length or truncated gene affected abscess formation, as it significantly reduced the pathogenicity of clone B2^p^. At least ten genes encoding alcohol dehydrogenases are found in the genome of *E*. *histolytica*. Two of them show 79% (EHI_192470) and 70% (EHI_198760) amino acid sequence identity with EHI_088020. The expression levels of both genes were similar in different clones. Interestingly, EHI_198760 (EhADH3) was described to be present in lower amounts in the non-pathogenic isolate Rahman, as well as in *E*. *dispar* in comparison to E. histolytica HM-1:IMSS [10, 58]. However, no correlation between EhAH3 amount and pathogenicity was observed [58].

Interestingly, none of the three genes (EHI_026360, EHI_056490, EHI_039020) up-regulated in the two non-pathogenic clones A1^np^ and B8^np^ vs. pathogenic clone B2^p^ affected ALA formation during infection with their overexpressing B2^p^ transfectants.

Of the genes that were more highly expressed in clone B2^p^ in comparison with clones A1^np^ or B8^np^, the only one impacting ALA formation when overexpressed was EHI_127670. EHI_127670 was one of the genes that was more highly expressed in clone B2^p^ than in the two non-pathogenic clones A1^np^ and B8^np^. Transfectants of either clone ectopically expressing this gene were able to induce ALA formation. However, since the pathogenic phenotype was only observed in 4/9 animals infected with A1^np^-EHI_127670 transfectants and 6/9 animals infected with B8^np^-EHI_127670 transfectants, these results were not statistically significant. Interestingly, if the results of both non-pathogenic clones overexpressing EHI_12760 were summarised the effect on ALA formation became significant. EHI_127670 encodes a putative protein of 111 amino acids. No homologues and no conserved domains within the protein were identified.

In this study we analysed the influence on ALA formation for 20 out of the 89 differentially expressed genes identified. However, it was not possible to overexpress 4 *rab* protein encoding genes (EHI_082070/*ehrab7d*, EHI_169280/*ehrab7e*, EHI_187090/*ehrab7g*, EHI164900), which are highly expressed in clone A1^np^ and very low expressed in clone B2^p^. This differential expression was also observed comparing the non-clonal cell lines A and B [12]. Rab GTPases are essential for the regulation of vesicular trafficking in the endocytic and exocytic/secretory pathways of eukaryotic cells [59]. The genome of *E*. *histolytica* contains more than 90 *rab* genes, including nine of the Rab7 isotype. Therefore, *E*. *histolytica* seems to be an organism with extremely diverse and complex Rab functions [60, 61]. One of the Rab7 isotypes, namely EhRab7A, is involved in transport of CPs to phagosomes and in recycling of a CP receptor from the phagosomes to the *trans*-Golgi network [62–64]. EhRab7A and EhRab7B are involved in lysosome biogenesis [61]. There is additional evidence that all EhRab7 isotypes are sequentially and coordinately involved in phagosome biogenesis [61]. However, so far it remains elusive whether the differential expression of the 4 *rab* genes indeed influences the ALA formation.

### Conclusion

In this study, no correlation was found between the ability of *E*. *histolytica* clones to produce amoebic liver abscesses and their cysteine protease, haemolytic, erythrophagocytosis, or cytopathic activities, or their sizes or growth characteristics. However, the clones showed different expression profiles. We conclude that different mechanisms result in the loss of *E*. *histolytica* pathogenicity, because only a few genes were found to be differentially regulated in the same way when either of the two non-pathogenic clones A1^np^ and B8^np^ were compared with the pathogenic clone B2^p^. However, overexpression of seven different genes, encoding a metallopeptidase, C2 domain proteins, alcohol dehydrogenases, and hypothetical proteins in the pathogenic clone B2^p^ correlated with reduced ability of *E*. *histolytica* to produce amoebic liver abscesses. Only one gene was identified whose overexpression transformed a non-pathogenic phenotype into a pathogenic one.

## Methods

### Ethics statement

Animal experiments were carried out in accordance with the guidelines from the German National Board for Laboratory Animals and ARRIVE guidelines (https://www.nc3rs.org.uk/arrive-guidelines) and approved by the review board of the State of Hamburg, Germany (Ministry of Health and Consumer Protection/Behörde für Gesundheit und Verbraucherschutz—ethical permits 145/13, 20.01.2014)

### *E*. *histolytica* cell culture

*E*. *histolytica* trophozoites were cultured axenically in TYI-S-33 medium in plastic tissue culture flasks [65]. *E*. *histolytica* cell lines HM-1:IMSS-A and HM-1:IMSS-B were derived from the isolate HM-1:IMSS and both were originally obtained from the American Type Culture Collection (ATCC) under the catalogue number 30459[11]. HM-1:IMSS was originally isolated from a colonic biopsy of rectal ulcer from an adult male patient with amoebic dysentry in 1967 (Mexico City, Mexico). The monoxenic cultured HM-1:IMSS isolate was passed from Margarita de la Torre to Louis S. Diamond who adapted it to axenic cultivation. Thereafter, this axenically cultivated HM-1:IMSS isolate was transferred to the ATCC library. Cell line A was sent to us in 2001 by Barbara Mann (Charlottesville, University of Virginia), as a batch of cells from the same culture that was used for DNA preparation to sequence the *E*. *histolytica* genome [66]. The pathogenic cell line B was obtained directly from ATCC in 1991. Since then, the ability of cell line B to induce liver pathology remained stable.

Both cell lines were cloned by limited dilution. For this, a dilution of 120 amoebae/24 ml TYI-S-33 medium was prepared and 200 μl of this dilution was added to each well of a 96-well plate. The presence of only one amoebae/well was analysed microscopically and the trophozoites were cultivated under anaerobic conditions using Anaerocult (Merck) for one week. Afterwards the clones were transferred for further cultivation to tissue culture flasks.

For individual experiments, 1 × 10^6^ trophozoites were cultivated for 24 h in 75 mL culture flasks. Subsequently, after chilling on ice for 5 min, trophozoites were harvested by sedimentation at 430 × *g* at 4°C for 5 min. The resulting cell pellets were washed twice either in phosphate-buffered saline (PBS; 6.7 mM NaHPO_4_, 3.3 mM NaH_2_PO_4_, 140 mM NaCl, pH 7.2) or in incomplete TYI-S-33 medium (medium without serum). To prepare amoeba extracts, cells were lysed over four freeze-thaw cycles in CO_2_/ethanol and sedimented by centrifugation (9000 × *g* at 4°C for 15 min).

### ALA formation in gerbils and mice

Animal infections were performed with 10- to 12-week-old female gerbils obtained from JANVIER LABS (Saint Berthevin Cedex 53941 France) or with 10- to 12-week-old C57BL/6 male mice bred in the animal facility of the Bernhard Nocht Institute for Tropical Medicine, Hamburg, Germany. All animals were maintained in a specific pathogen-free environment. Animal experiments were approved by the review board of the State of Hamburg, Germany (Ministry of Health and Consumer Protection/Behörde für Gesundheit und Verbraucherschutz, (145/13, 20.01.2014) and conducted in accordance with institutional and ARRIVE guidelines (https://www.nc3rs.org.uk/arrive-guidelines).

For gerbil infections, 1 × 10^6^ trophozoites in 50 μL of incomplete TYI-S-33 medium (without serum) were injected into the left liver lobe, as described previously [67]. For mice infection experiments, 1.25 × 10^5^ trophozoites in 25 μL of incomplete TYI-S-33 medium were injected into the liver, as described by Lotter and colleagues [23].

To analyse ALA formation of the various amoeba clones, gerbils were sacrificed at 7 days post infection and the extent of the abscessed liver area was measured manually using a caliper and determined as size in mm^2^. For each *E*. *histolytica* clone, ALA formation was analysed in at least four animals. Significance (*p*-values) was established using the Mann-Whitney *U* test.

MRI was performed to analyse the time course of ALA formation using a small animal 7 Tesla MR scanner (ClinScan, Bruker Biospin GmbH, Ettlingen, Germany). For these experiments, gerbil and mouse livers were imaged *in vivo* on days 3, 5, 7 and 10 after intrahepatic injection of *E*. *histolytica*. Anaesthesia was performed as described by Ernst and colleagues [68]. Images were acquired using T2-weighted fast spin echo (T2w FSE) sequences for high-resolution anatomical reference. Total abscess volume was calculated by measuring the region of interest (ROI) in each abscess-containing slice, using transversal sections of the abdomen and the OsiriX Imaging Software DICOM Viewer (Open-source version 32-bit 4.1.1). Significance (*p-*values) was established using an unpaired t test.

### RNA extraction and qRT-PCR

*E*. *histolytica* trophozoites (1 × 10^6^) were cultivated in 75 mL culture flasks for 24 h. The cells were harvested after chilling on ice for 5 min and sedimented at 200 × *g* for 5 min at 4°C. Cell pellets were washed twice with PBS. To isolate total RNA, trophozoites were treated with TRIzol reagent (Thermo Fisher Scientific, Schwerte, Germany) following the manufacturer’s instructions. Extracted RNA was further purified using the RNeasy mini kit (Qiagen, Hilden, Germany) but without β-mercaptoethanol in buffer RLT, and DNA was digested with DNase I (Qiagen, Hilden, Germany). Total RNA for transcriptomics analyses was purified using the *mir*Vana miRNA isolation kit (Ambion-Thermo Fisher Scientific, Schwerte, Germany), according to the manufacturer’s instructions.

cDNA synthesis was obtained using the SuperScript III Reverse Transcriptase system (Thermo Fisher Scientific, Schwerte, Germany). Briefly, RNase-free and DNase-treated total RNA (1 μg in a 20 μL final volume) was mixed with 5 × First-Strand buffer, 1 mM dNTPs, 500 nM OdT-T71 (5′-GAG AGA GGA TCC AAG TAC TAA TAC GAC TCA CTA TAG GGA GAT_24_), 2 mM DTT, 0.5 mM MgCl_2_, 40 U RNaseOut (Thermo Fisher Scientific, Schwerte, Germany) and SuperScript III (200 U/μL). cDNA was synthesised for 1 h at 42°C.

For qRT-PCR experiments, sense and antisense primers were designed to amplify 100–120 bp fragments of the respective genes (S8 and S9 Tables). Quantitative amplifications were performed in Rotor-Gene PCR apparatus (Qiagen, Hilden, Germany) using RealMasterMix SYBR ROX Kit (5PRIME, Hilden, Germany). cDNA (1 μL) was mixed with 2.5 × RealMasterMix/20 × SYBR and 5 pmol/μL appropriate sense and antisense primers, to a final 20 μL volume. Amplification conditions were as follows: 40 cycles of 95°C for 15 s, 58°C for 20 s and 68°C for 20 s, and an adjacent melting step (67–95°C). Two biological replicates were analysed in duplicate. Relative differences in gene expression were calculated using the 2^-ΔΔCT^ method with Rotor-Gene software [69]. Depending on the experiment, clone A1^np^, clone B2^p^, or clone B8^np^ was used as the calibrator (= 1), and actin was used as the house-keeping gene for normalisation.

### Transcriptome analyses and bioinformatics

RNA for RNA-Seq library preparation was purified as described above. RNA quantity and quality were evaluated spectrophotometrically (NanoDrop 2000, Thermo Fisher Scientific, Schwerte, Germany) and with an Agilent 2100 Bioanalyzer with RNA 6000 Pico Assays kit (Agilent Technologies, Waldbronn, Germany).

Samples were Turbo DNase-treated using TURBO DNA-free Kit (Ambion-Thermo Fisher Scientific, Schwerte, Germany). After quality control, rRNA was depleted using RiboZero Magnetic Gold kit (Human/Mouse/Rat; Epicentre-Illumina, Munich, Germany) and Agencourt RNAClean XP kit (Beckmann Coulter, Krefeld, Germany), according to the manufacturers’ protocol. RNA-Seq libraries were then generated using ScriptSeq v2 kit (Epicentre-Illumina, Munich, Germany) according to the manufacturer’s instructions. Each library was indexed with Illumina-compatible barcodes to allow multiplexing. The individual libraries were assessed using Qubit dsDNA high sensitivity kit and Bioanalyzer DNA HS chips to ascertain the concentration (4 nM) and fragment size distribution, respectively, prior to library multiplexing. Libraries were denatured and diluted to final concentration of 8 pM for sequencing on the MiSeq platform following the manufacturer’s instructions. Reads were aligned to *E*. *histolytica* transcriptome (AmoebaDB 28, released 30 March 2016) using Bowtie 2 version 2.2.3 [70] and differential expression was analysed using DESeq [71] version 1.18.

### Determination of sizes, growth rates and motility of *E*. *histolytica* clone A1^np^, B2^p^ and B8^np^

To determine amoeba size, the circumference of 80 trophozoites of each clone was measured using a BZ9000 Keyence microscope (Keyence, Neu-Isenburg, Germany). To determine growth rate, 500 trophozoites of each clone were seeded into a 24-well plate and the cells were counted every 24 h over 72 h. The growth rate was determined three times in triplicate for each clone. The movement of the amoebae was directly filmed over a time period of 10 min using Evos FL Auto microscope from Life Technologies. A picture was taken every 5 sec. The movement of 80 amoebae/ clone was analysed manually using ImageJ version 2.0.0-rc-43/1.51d with plugins for manual tracking and chemotaxis. Significance (*p-*values) was established using the Mann-Whitney *U* test.

### Cysteine peptidase assay

CP activity was measured using the synthetic peptide Z-Arg-Arg-pNA (Bachem, Bubendorf, Switzerland) as substrate [72]. One unit of enzymatic activity is defined as the amount of protein that catalyses the generation of 1 μmoL *p*-nitroaniline per min.

### Substrate gel electrophoresis

Substrate gel electrophoresis was performed as described previously [73]. Briefly, amoebic extract (2 μg) was incubated in Laemmli buffer with 20 mM DTT, for 5 min at 37°C. For the substrate gel, 12% SDS-polyacrylamide gel was co-polymerised with 0.1% gelatine. After protein separation and incubation in buffer A (2.5% Triton X-100; 1 h) and buffer B (100 mM sodium acetate, pH 4.5, 1% Triton X-100, 20 mM DTT; 3 h), at 37°C, the gel was stained with Coomassie blue.

### Erythrophagocytosis assay

Erythrophagocytosis assay was performed as described by Biller and colleagues [11]. Human 0+ erythrocytes were provided by the blood bank of the University Medical Center Hamburg-Eppendorf (UKE)–Transfusion Medicine–Germany. Human erythrocytes and trophozoites were washed twice with serum-free TYI-S-33 medium. Erythrocytes and amoebae were mixed at a 1000:1 ratio (2 × 10^8^ erythrocytes, 2 × 10^5^ amoebae), to a final volume of 400 μL, in serum-free TYI-S-33 medium and incubated in parallel at 37°C for 30 min. To stop phagocytosis and lyse non-phagocytosed erythrocytes, 1 mL of distilled water was added, twice. Trophozoites were washed twice with PBS. Average numbers of ingested erythrocytes were quantified by measuring the absorbance at 397 nm after trophozoite lysis in 90% formic acid. The experiment was performed three times in triplicate. Significance (*p*-values) was established using the Mann-Whitney *U* test.

### Haemolytic activity assay

Haemolytic activity assay was performed as described by Biller and colleagues [11]. Human erythrocytes and trophozoites were washed three times with PBS. The assay was performed by mixing trophozoites and erythrocytes in a 1:2000 ratio (2 × 10^5^ amoebae with 4 × 10^8^ erythrocytes per mL of PBS), followed by incubation for 1 h at 37°C. After incubation, the cells were sedimented for 1 min at 2000 × *g*. Haemoglobin released into the supernatant was measured at 570 nm in a spectrophotometer. Separately incubated erythrocytes and trophozoites were used as negative controls. To determine 100% haemoglobin release, 4 × 10^8^ erythrocytes were lysed in 1 mL of water. The experiment was performed three times in triplicate. Significance (*p*-values) was established using the Mann-Whitney *U* test.

### Cytopathic activity

Interaction of trophozoites and Chinese hamster ovarian (CHO) cells was determined by a modified method of Bracha and Mirelman [74]. CHO cells defective in glycosaminoglycan biosynthesis (CHO-745; American Type Culture Collection No. CRL-2242) were used. CHO cells (1 × 10^5^ per well) were grown for 24 h in 24-well plates in Ham’s F12 (with l-Glutamine) medium supplemented with 10% fetal calf serum (FCS) and penicillin-streptomycin. After washing the CHO cells with preheated (37°C) Ham’s medium, 500 μL of Ham’s medium was added. *E*. *histolytica* trophozoites (1 × 10^5^) were washed twice with serum-free TYI-S-33 medium, resuspended in 500 μL of ABS-free TYI-S-33 and added to the CHO cells. The mixture was incubated for 20 min at 37°C under 5% CO_2_. Cells were washed with 1 mL of ice-cold PBS and treated with 0.5 mL of 4% paraformaldehyde in PBS for 2 min. After another PBS wash, the cells were stained with 500 μL of 0.1% methylene blue for 2 min. Finally, the cells were washed with 0.01% methylene blue and PBS. Cells were lysed with 1 mL of 0.1 M HCl for 30 min at 37°C. Samples were photometrically analysed at 660 nm. As a control, methylene blue concentration was determined for CHO cells that had not been co-cultivated with trophozoites (i.e., no destruction of cell monolayer). Experiments were performed three times in sextuplicate. Significance (*p*-values) was established using the Mann-Whitney *U* test.

### Expression constructs

All plasmids used for *E*. *histolytica* trophozoite transfections are derivatives of the expression vector pEhNEO/CAT (pNC) [75, 76]. Genes of interest were amplified by PCR using genomic *E*. *histolytica* DNA as a template, cloned into TOPO TA vector, sequenced and cloned into pNC using *Kpn*I and *Bam*HI restriction sites (S9 Table). For overexpression, coding sequences of the genes of interest were flanked by 485 bp 5′-untranslated sequence of the *E*. *histolytica* lectin gene and 600 bp 3′-untranslated region of the actin gene. Neomycin phosphotransferase was used as a selectable marker.

Transfections were performed by electroporation as described previously [76]. Two days post transfection, cells were transferred to a selection medium containing 10 μg/mL G-418 sulphate, for approximately 2 weeks. Subsequently, the cells were cloned by a limited dilution method and cultivated in the presence of 20 μg/mL G418. Successful overexpression of at least four clones was checked by qRT-PCR. For infection experiments, trophozoites were cultivated for 24 h in the absence of G418.

## Supporting Information

S1 Table Summary of *p*-values. (DOC)

S2 Table Comparison of the transcriptomes of clone A1^np^ and clone B2^p^. (XLS)

S3 Table Comparison of the transcriptomes of clone B8^np^ and clone B2^p^. (XLS)

S4 Table Characteristics of the proteins encoded by the genes found to be differentially expressed between clone A1^np^ and clone B2^p^ and/or clone B8^np^ and clone B2^p^. (DOC)

S5 Table Relative expression of overexpressing genes in clone B2^p^ transfectants that originally showed higher expression in clone A1^np(a)^ and/or B8^np(b)^ than in clone B2^p^. (DOC)

S6 Table Relative expression of overexpressing genes in clone A1^np^ transfectants that originally showed higher expression in clone B2^p^ than in clone A1^np^. (DOC)

S7 Table Relative expression of overexpressing genes in clone B8^np^ transfectants that originally showed higher expressed in clone B2^p^ than in clone B8^np^. (DOC)

S8 Table Oligonucleotides used for verification of transcriptome results using qPCR. (DOCX)

S9 Table Oligonucleotides for gene amplification and for the analysis of overexpression in transfectants using qPCR. (DOCX)
