# Supplementary material for: Overexpression of Differentially Expressed Genes Identified in Non-pathogenic and Pathogenic Entamoeba histolytica Clones Allow Identification of New Pathogenicity Factors Involved in Amoebic Liver Abscess Formation
Source: PLoS Pathog. 2016 Aug 30;12(8):e1005853. doi: 10.1371/journal.ppat.1005853 (PMC5004846; doi:10.1371/journal.ppat.1005853)
Supplement: S1 Table — (DOC) [file ppat.1005853.s001.doc]

**S1 Table.** Summary of *p*-values

| Control | Sample | *p*-value |  |
| --- | --- | --- | --- |
| **Fig 1** |  |  |  |
| HM-1:IMSS-A | A1 | >0.9999 | ns^1^ |
| HM-1:IMSS-A | A2 | >0.9999 | ns |
| HM-1:IMSS-A | A3 | >0.9999 | ns |
| HM-1:IMSS-A | A4 | >0.9999 | ns |
| HM-1:IMSS-A | A5 | >0.9999 | ns |
| HM-1:IMSS-A | A6 | 0.4286 | ns |
| HM-1:IMSS-A | A7 | >0.9999 | ns |
| HM-1:IMSS-A | A8 | >0.9999 | ns |
| HM-1:IMSS-A | A9 | >0.9999 | ns |
| HM-1:IMSS-A | A10 | >0.9999 | ns |
| HM-1:IMSS-A | A11 | 0.4286 | ns |
| HM-1:IMSS-A | A12 | 0.4286 | ns |
| HM-1:IMSS-B | B1 | 0.0286 | * |
| HM-1:IMSS-B | B2 | 0.2141 | ns |
| HM-1:IMSS-B | B3 | 0.0571 | ns |
| HM-1:IMSS-B | B4 | 0.2286 | ns |
| HM-1:IMSS-B | B5 | 0.7429 | ns |
| HM-1:IMSS-B | B6 | 0.0571 | ns |
| HM-1:IMSS-B | B7 | 0.1429 | ns |
| HM-1:IMSS-B | B8 | 0.0079 | ** |
| HM-1:IMSS-B | B9 | 0.4 | ns |
| HM-1:IMSS-B | B10 | 0.4286 | ns |
| HM-1:IMSS-B | B11 | 0.0286 | * |
| HM-1:IMSS-B | B12 | 0.0286 | * |
|  |  |  |  |
| **Fig 2** |  |  |  |
| B8^np^ | B8_1 | >0.9999 | ns |
| B8^np^ | B8_2 | >0.9999 | ns |
| B8^np^ | B8_3 | >0.9999 | ns |
| B8^np^ | B8_4 | >0.9999 | ns |
| B8^np^ | B8_5 | >0.9999 | ns |
| B2^p^ | B2_1 | 0.2082 | ns |
| B2^p^ | B2_1 | 0.4235 | ns |
| B2^p^ | B2_1 | 0.9806 | ns |
| B2^p^ | B2_1 | 0.3217 | ns |
| B2^p^ | B2_1 | 0.9223 | ns |
|  |  |  |  |
| **Fig 3A** |  |  |  |
| HM-1:IMSS-A | A1 | 0.9864 | ns |
| HM-1:IMSS-A | A2 | 0.4593 | ns |
| HM-1:IMSS-A | A3 | 0.0112 | * |
| HM-1:IMSS-A | A4 | 0.5303 | ns |
| HM-1:IMSS-A | A5 | 0.6747 | ns |
| HM-1:IMSS-A | A6 | 0.6195 | ns |
| HM-1:IMSS-A | A7 | 0.5294 | ns |
| HM-1:IMSS-A | A8 | 0.3769 | ns |
| HM-1:IMSS-A | A9 | 0.6746 | ns |
| HM-1:IMSS-A | A10 | 0.0087 | ** |
| HM-1:IMSS-A | A11 | 0.0859 | ns |
| HM-1:IMSS-A | A12 | 0.0117 | ns |
| HM-1:IMSS-B | B1 | 0.0357 | * |
| HM-1:IMSS-B | B2 | 0.7574 | ns |
| HM-1:IMSS-B | B3 | 0.4845 | ns |
| HM-1:IMSS-B | B4 | 0.0050 | ** |
| HM-1:IMSS-B | B5 | 0.0006 | *** |
| HM-1:IMSS-B | B6 | 0.0342 | * |
| HM-1:IMSS-B | B7 | 0.0038 | ** |
| HM-1:IMSS-B | B8 | 0.0016 | ** |
| HM-1:IMSS-B | B9 | 0.1497 | ns |
| HM-1:IMSS-B | B10 | 0.9779 | ns |
| HM-1:IMSS-B | B11 | 0.1482 | ns |
| HM-1:IMSS-B | B12 | 0.6123 | ns |
|  |  |  |  |
| **Fig 5A** |  |  |  |
| Control | EHI_015290 | 0.0016 | ** |
| Control | EHI_042870 | 0.0007 | *** |
| Control | EHI_082070 | 0.5405 | ns |
| Control | EHI059860_ | 0.0442 | * |
| Control | EHI_118130 | 0.8412 | ns |
| Control | EHI_075690 | 0.0382 | * |
| Control | EHI_048140 | 0.6109 | ns |
| Control | EHI_058920 | 0.0227 | * |
| Control | EHI_088020 | 0.0129 | * |
| Control | EHI_151930 | 0.1164 | ns |
| Control | EHI_160670 | <0.0001 | **** |
| Control | EHI_180390 | 0.3496 | ns |
| Control | EHI_026360 | 0.3061 | ns |
| Control | EHI_056490 | 0.0822 | ns |
| Control | EHI_039020 | 0.6341 | ns |
|  |  |  |  |
| **Fig 5B** |  |  |  |
| Control | EHI_014170 | >0.9999 | ns |
| Control | EHI_127670 | 0.2927 | ns |
| Control | EHI_144490 | 0.4444 | ns |
| Control | EHI_144610 | >0.9999 | ns |
|  |  |  |  |
| **Fig 5C** |  |  |  |
| Control | EHI_127670 | 0.0589 | ns |
| Control | EHI_073680 | 0.4071 | ns |
| Control | EHI_14490 | 0.6737 | ns |
| Control | EHI_144610 | >0.9999 | ns |
|  |  |  |  |
| **Fig 5D** |  |  |  |
| Control | EHI_127670 | 0.0263 | * |
| Control | EHI_14490 | 0.3625 | ns |
| Control | EHI_144610 | 0.7214 | ns |

ns^1^: Not significant
