# Supplementary material for: Overexpression of Differentially Expressed Genes Identified in Non-pathogenic and Pathogenic Entamoeba histolytica Clones Allow Identification of New Pathogenicity Factors Involved in Amoebic Liver Abscess Formation
Source: PLoS Pathog. 2016 Aug 30;12(8):e1005853. doi: 10.1371/journal.ppat.1005853 (PMC5004846; doi:10.1371/journal.ppat.1005853)
Supplement: S4 Table — (DOC) [file ppat.1005853.s004.doc]

**S4 Table** Characteristics of the proteins encoded by the genes found to be differentially expressed between clone A1^np^ and clone B2^p^ and/or clone B8^np^ and clone B2^p^.

| Gene ID | Protein name | Family | Microarray (> 3 fold)^1^ | | Surface proteome^2^ | SP^3^ | TM | Domain |
| --- | --- | --- | --- | --- | --- | --- | --- | --- |
|  |  |  | A>B | B>A |  |  |  |  |
| EHI_015290 | C2 domain protein (EhC2-3) | C2 superfamily |  |  | x |  |  |  |
| EHI_042870 | cell surface protease gp63 (EhMP8-2) | peptidase M8 superfamily | 21.44 |  |  | x | 1 |  |
| EHI_082070 | Rab family GTPase (EhRab7D) | small GTPase superfamily | 25.37 |  | x |  |  |  |
| EHI_059860 | C2 domain-containing protein (EhC2-5) | C2 superfamily |  |  |  |  |  |  |
| EHI_118130 | C2 domain-containing protein (EhC2-2) | C2 superfamily | 6.62 |  | x |  |  |  |
| EHI_169280 | Rab family GTPase (EhRab7E) | small GTPase superfamily | 62 |  | x |  |  |  |
| EHI_074080 | hypothetical protein |  | 52.11 |  | x |  |  |  |
| EHI_187090 | Rab family GTPase (EhRab7G) | small GTPase superfamily | 14.5 |  |  |  |  |  |
| EHI_155060 | chaperone ClpB | ClpA/B family |  |  |  |  |  |  |
| EHI_005657 | hypothetical protein | ClpA/B family |  |  |  |  |  |  |
| EHI_151890 | EF-hand calcium-binding domain-containing protein |  |  |  |  |  |  | EF-hand domain |
| EHI_026360 | phosphoserine aminotransferase (EhPSAT) |  |  |  |  |  |  |  |
| EHI_075660 | CAAX prenyl protease (EhCAAX) |  |  |  | x |  | 7 |  |
| EHI_097480 | hypothetical protein |  |  |  |  |  |  |  |
| EHI_075690 | hypothetical protein |  | 3.46 |  |  |  | 4 | tetraspanin/peripherin |
| EHI_137080 | hypothetical protein |  |  |  |  |  | 2 |  |
| EHI_075640 | protein phosphatase domain-containing protein | protein phosphatase 2C family |  |  |  |  |  |  |
| EHI_056490 | 20 kDa antigen |  |  |  |  |  |  |  |
| EHI_075700 | casein kinase II regulatory subunit family protein |  | 3.09 |  |  |  |  |  |
| EHI_164900 | Rab family GTPase | small GTPase superfamily |  |  | x |  |  |  |
| EHI_114950 | hypothetical protein | septin |  |  |  |  |  | AIG1-type guanine nucleotide-binding domain |
| EHI_039020 | actobindin |  |  |  | x |  |  | WH2 domain |
| EHI_086690 | hypothetical protein |  | 3 |  |  |  |  |  |
| EHI_175930 | hypothetical protein |  |  |  |  | x |  | MHCK/EF2 kinase |
| EHI_086570 | hypothetical protein |  |  |  |  |  |  |  |
| EHI_028920 | heat shock protein |  |  |  |  |  | 1 | uncharacterized protein family UPF0592 |
| EHI_156560 | heat shock protein | ClpA/B family |  |  |  |  |  |  |
| EHI_126560 | AIG1 family protein |  |  |  |  |  |  |  |
| EHI_184500 | hypothetical protein |  |  |  |  |  |  | DOCK1 homologue |
| EHI_171750 | cysteine synthase 2 |  |  |  |  |  |  | tryptophan synthase beta subunit-like PLP dependent enzyme |
| EHI_126550 | AIG1 family protein |  |  |  |  |  |  | AIG1-type guanine nucleotide-binding domain |
| EHI_034710 | heat shock protein | ClpA/B family |  |  |  |  |  |  |
| EHI_006170 | eukaryotic translation initiation factor 6 |  |  |  | x |  |  |  |
| EHI_085470 | splicing factor3B subunit 1 |  |  |  |  |  | 2 | armadillo-type fold |
| EHI_148550 | protein tyrosine kinase domain-containing protein |  |  |  |  |  | 1 | protein kinase-like domain |
| EHI_042860 | heat shock protein | ClpA/B family |  |  |  |  |  |  |
| EHI_037550 | hypothetical protein |  |  |  |  |  |  |  |
| EHI_123830 | DNA mismatch repair protein Msh2 |  |  |  |  |  |  |  |
| EHI_060340 | cysteine synthase A |  |  |  |  |  |  |  |
| EHI_022620 | heat shock protein | ClpA/B family |  |  |  |  |  |  |
| EHI_169470 | fructose-1,6-bisphosphate aldolase |  |  |  |  |  |  |  |
| EHI_086520 | 3' exoribonuclease family protein |  | 3.14 |  |  |  |  | ribosomal S5 domain |
| EHI_177570 | RNA 3'-terminal phosphate cyclase |  |  |  |  |  |  |  |
| EHI_116830 | d-phosphoglycerate dehydrogenase |  |  |  |  |  |  | NAD(P) binding domain |
| EHI_075710 | hypothetical protein |  |  |  |  |  |  |  |
| EHI_086540 | replication factor C subunit 4 | P-loop containing nucleoside triphosphate hydrolase | 3.02 |  | x |  |  |  |
| EHI_127670 | hypothetical protein |  |  |  |  |  |  |  |
| EHI_144490 | hypothetical protein | septin |  |  |  |  |  | AIG1-type guanine nucleotide-binding domain |
| EHI_169670 | hypothetical protein |  |  | 14.29 |  |  |  | EEIG1/EHBP1 N-terminal domain, DILUTE domain |
| EHI_050490 | hypothetical protein |  |  |  |  |  |  |  |
| EHI_062080 | hypothetical protein |  |  |  |  |  |  |  |
| EHI_178610 | tyrosine kinase |  |  |  |  | x | 2 | protein kinase domain |
| EHI_013240 | hypothetical protein |  |  | 3.44 |  |  |  |  |
| EHI_076370 | hypothetical protein |  |  |  |  |  |  |  |
| EHI_121820 | hypothetical protein | [quinoprotein alcohol dehydrogenase-like superfamily](http://www.ebi.ac.uk/interpro/entry/IPR011047) |  | 5.26 |  | x | 1 | BEACH domain |
| EHI_111330 | hypothetical protein |  |  |  |  |  |  |  |
| EHI_192510 | hypothetical protein | heat shock protein 70 family |  |  |  |  |  | heat shock protein 70, peptide binding domain |
| EHI_017760 | tyrosine kinase |  |  |  |  |  | 2 | protein kinase-like domain, Growth factor receptor cysteine rich domain, EGF-like domain, Furin like repeat |
| EHI_165190 | hypothetical protein |  |  | 8.33 |  |  |  |  |
| EHI_014170 | hypothetical protein |  |  | 5.26 |  |  |  | EEIG1/EHBP1 N-terminal domain, DILUTE domain |
| EHI_101630 | hypothetical protein | septin |  |  |  |  |  | AIG1-type guanine nucleotide-binding domain |
| EHI_144610 | methionine gamma-lyase | [pyridoxal phosphate-dependent transferas](http://www.ebi.ac.uk/interpro/entry/IPR015424)e |  |  |  |  |  |  |
| EHI_029360 | hypothetical protein |  |  |  |  |  |  |  |
| EHI_057550 | methionine gamma-lyase | [pyridoxal phosphate-dependent transferas](http://www.ebi.ac.uk/interpro/entry/IPR015424)e |  |  |  |  |  |  |
| EHI_029380 | thioredoxin | thioredoxin family |  |  |  |  |  | thioredoxin domain |
| EHI_176590 | AIG1 family protein | septin |  |  |  |  |  | AIG1-type guanine nucleotide-binding domain |
| EHI_150770 | heat shock protein 70 | heat shock protein 70 family |  |  |  |  |  | heat shock protein 70, peptide binding domain |
| EHI_197980 | Myb family DNA-binding protein |  |  |  |  |  |  | Myb domain |
| EHI_104570 | ubiquitin ligase |  |  |  |  |  |  | HECT domain |
| EHI_063550 | Myb-like DNA-binding domain-containing protein |  |  |  |  |  |  | Myb domain |
| EHI_062970 | hypothetical protein |  |  |  |  |  |  |  |
| EHI_087000 | hypothetical protein |  |  |  |  |  | 4 |  |
| EHI_033700 | RecF/RecN/SMC domain-containing protein |  |  |  |  |  |  | P-loop containing nucleoside triphosphate hydrolase |
| EHI_063580 | hypothetical protein |  |  |  |  |  |  |  |
| EHI_131490 | leucine-rich repeat protein, BspA family | BspA family |  |  |  |  |  | leucine-rich repeat domain |
| EHI_020250 | lecithin:cholesterol acyltransferase domain-containing protein |  |  |  |  |  |  | lecithin:cholesterol acyltransferase domain |
| EHI_062960 | hypothetical protein |  |  | 9.09 |  | x | 1 |  |
| EHI_183400 | galactose-inhibitable lectin 35 kDa subunit |  |  |  |  | x |  |  |
| EHI_048140 | hypothetical protein |  |  |  |  |  | 1 |  |
| EHI_058920 | hypothetical protein |  |  |  |  | x |  |  |
| EHI_037160 | hypothetical protein |  |  |  |  |  |  | Zinc finger, LIM type |
| EHI_088020 | alcohol dehydrogenase | alcohol dehydrogenase, iron-type |  |  | x |  |  |  |
| EHI_151930 | hypothetical protein |  |  |  |  |  |  | HAD-like domain |
| EHI_160670 | alcohol dehydrogenase 3 | alcohol dehydrogenase, iron-type |  |  |  |  |  |  |
| EHI_180390 | AIG1 family protein |  |  | 14.29 |  |  | 1 | AIG1-type guanine nucleotide-binding domain |
| EHI_073680 | leucine-rich repeat-containing protein |  |  |  |  |  |  | leucine-rich repeat domain |
| EHI_062080 | hypothetical protein |  |  |  |  |  |  |  |
| EHI_191730 | hypothetical protein |  |  |  |  | x | 1 |  |

^1^ Biller et al., 2010; ^2^ Biller et al., 2014; ^3^Signal peptide (SP); 4 Number of transmembrane domains (TM)
