# Supplementary material for: Overexpression of Differentially Expressed Genes Identified in Non-pathogenic and Pathogenic Entamoeba histolytica Clones Allow Identification of New Pathogenicity Factors Involved in Amoebic Liver Abscess Formation
Source: PLoS Pathog. 2016 Aug 30;12(8):e1005853. doi: 10.1371/journal.ppat.1005853 (PMC5004846; doi:10.1371/journal.ppat.1005853)
Supplement: S5 Table — (DOC) [file ppat.1005853.s005.doc]

**S5 Table** Relative expression of overexpressing genes in clone B2^p^

transfectants that originally showed higher expression in clone A1^np(a)^

and/or B8^np(b)^ than in clone B2^p^.

| Clone B2^p^ transfectant | Name (Abbreviation) | Relative expression  (ddCT method)* |
| --- | --- | --- |
| pNC (control) |  | 1 |
| pNC: EHI_015290 | EhC2-3^a^ | 2.4 |
| pNC: EHI_042870 | EhMP8-2^a^ | 5.85 |
| pNC: EHI_082070 | EhRab7D^a^ | 5.73 |
| pNC: EHI_059860 | EhC2-5^a^ | 5.82 |
| pNC: EHI_118130 | EhC2-2^a^ | 2.76 |
| pNC: EHI_075690 | HypProt^a^ | 66.00 |
| pNC: EHI_169280 | EhRab7E^a^ | 0.66 |
| pNC: EHI_075660 | EhCAXX^a^ | 1.04 |
| pNC: EHI_074080 | HypProt^a^ | 1.22 |
| pNC: EHI_187090 | EhRab7G^a^ | 1.4 |
| pNC:EHI_048140 | HypProt^b^ | 66.59 |
| pNC:EHI_058920 | HypProt^b^ | 4.33 |
| pNC:EHI_088020 | ADH^b^ | 25.2 |
| pNC:EHI_151930 | HypProt^b^ | 26.54 |
| pNC:EHI_160670 | ADH3^b^ | 133.17 |
| pNC:EHI_180390 | AIG1^b^ | 315.89 |
| pNC:EHI_026360 | EhPSAT^a/b^ | 124.20 |
| pNC: EHI_056490 | 20 kDa antigen^a/b^ | 234.52 |
| pNC: EHI_039020 | actobindin ^a/b^ | 111.08 |

*Ehactin was used as a normalizer
