# Supplementary material for: Overexpression of Differentially Expressed Genes Identified in Non-pathogenic and Pathogenic Entamoeba histolytica Clones Allow Identification of New Pathogenicity Factors Involved in Amoebic Liver Abscess Formation
Source: PLoS Pathog. 2016 Aug 30;12(8):e1005853. doi: 10.1371/journal.ppat.1005853 (PMC5004846; doi:10.1371/journal.ppat.1005853)
Supplement: S6 Table — (DOC) [file ppat.1005853.s006.doc]

**S6 Table** Relative expression of overexpressing genes in clone A1^np^

transfectants that originally showed higher expression in clone B2^p^

than in clone A1^np^.

| Clone A1^np^ transfectant | Name  (Abbreviation) | Relative expression  (ddCT method)* |
| --- | --- | --- |
| pNC (control) |  | 1 |
| pNC: EHI_127670 | HypProt | 36.56 |
| pNC: EHI_144490 | HypProt | 490.87 |
| pNC: EHI_169670 | HypProt | 1.39 |
| pNC: EHI_014170 | HypProt | 4.38 |
| pNC: EHI_144610 | Meth-g-lyse | 48.40 |

*Ehactin was used as a normalizer
