# Supplementary material for: Overexpression of Differentially Expressed Genes Identified in Non-pathogenic and Pathogenic Entamoeba histolytica Clones Allow Identification of New Pathogenicity Factors Involved in Amoebic Liver Abscess Formation
Source: PLoS Pathog. 2016 Aug 30;12(8):e1005853. doi: 10.1371/journal.ppat.1005853 (PMC5004846; doi:10.1371/journal.ppat.1005853)
Supplement: S8 Table — (DOCX) [file ppat.1005853.s008.docx]

**S8 Table** Oligonucleotides used for verification of transcriptome results using qPCR

| **Gene name** | **Gene ID** | **Oligonucleotide sequence (5’)** | **Oligonucleotide sequence (3’)** |
| --- | --- | --- | --- |
| *actin* | EHI_142730 | AAGCTGCATCAAGCAGTGAA | GGAATGATGGTTGGAAGAGG |
| EhC2-2 (C2 domain containing protein) | EHI_118130 | CCAGGTTATCCTCAACAACC | GTTGAGCTGGTACTCCTGGT |
| EhC2-3 (C2 domain containing protein) | EHI_015290 | AACAACAACCAGGTGCTTAGC | TCCTCCTTGTGGTGGATAGC |
| EhC2-5 (C2 domain containing protein) | EHI_059860 | AAGTGGTTGATACTTGGTTAGG | GGAGCACCAACTGTTAAGC |
| EhRab7D (Rab family GTPase) | EHI_082070 | GCAGGAAGACCAGCATTATT | GGTTATTGACGTATCGGTTG |
| EhRab7E (Rab family GTPase) | EHI_169280 | CCGTGGTAGTGATTGTTGTC | AACATTAGCACCATTGAGGAA |
| EhRab7G (Rab family GTPase) | EHI_187090 | CTCACGAGCACTCAATGGAA | TTCCTTCACACCATTGACGA |
| EhMP8-2 (cell surface protease gp63) | EHI_042870 | ATTGCTGTCATTCCTGTGTG | TCACGAACTTCTTCTGCTTG |
| CAAX prenyl protease | EHI_075660 | GGAAATGCATTCTTTTCAGGA | TGTTTACAATGTCCAACTTCATGT |
| Hypothetical protein | EHI_074080 | GGTTATCCACCACAACCAAT | CCATAGGACCTGACATTCCTC |
| Hypothetical protein | EHI_075690 | TGCTAGATCAGCTGAAGGAA | AATTGCACCAGCAACAATAA |
| Hypothetical protein | EHI_086690 | AACAAGCTGCTGATGACG | AGATATGCTTCAGCTTCTGC |
| EhC2-DIL1 hypothetical protein | EHI_169670 | CATTCAACCAGCAATTGAAGT | TGATGATTCAAGTTATCCGACT |
| EhC2-DIL2 hypothetical protein | EHI_014170 | GCAGATCTTAATGGTGCTTCAA | CTACCACCAGCCTCACCAAG |
| Hypothetical protein 6 (Beach domain) | EHI_121820 | ATTGAATTGTGCTGCTCGTC | TCACCAATTCCAATAGTTCCTT |
| Hypothetical protein 7 | EHI_013240 | CATGTTCTGGAGACCCAACA | CAAATTGATCAACTGGCATATC |
| Protein phosphatase domain-containing protein | EHI_075640 | TTCTGATGATACTCCAACACC | GCAATACATTGTCCTTCTGC |
| Casein kinase II regulatory subunit family protein | EHI_075700 | TGATAATCCAGGTATGGAACC | TCTTGAAGCGAATTGTTGTG |
| Replication factor C subunit 4 | EHI_086540 | GGTCAACCAGGAATTGGTAA | CAGAAGCATTCAGTTCCAGA |
| Hypothetical protein | EHI_127670 | AGAGAGCTACTCAACATGG | CATATTCATCACACTCTTCC |
| Tyrosine kinase | EHI_178610 | TTGCCTCGTTACAGAATATG | TAGCTGCGTCAATGACTAAT |
| Hypothetical protein | EHI_111330 | GCACAACGAGGAGTTGC | TACAATCACACGTCTTAACG |
| Hypothetical protein | EHI_165190 | TCTTATTCAACGCCCAGTTA | AGCTTCAACATCAATCCAGT |
| 20 kDa antigen | EHI_056490 | ATGAGAGCACGTATGGTAGG | ATGAAGAAGGTCCACAACC |
| Hypothetical protein | EHI_144490 | TATGCTTACATTCTGCGATG | TAAATACCATGGTGGGGTTA |
| Methionine gamma-lyase | EHI_144610 | ACATGCTTATCCAATCTTCC | CCAAGTCTAGAGTAGATATGACC |
| Hypothetical protein | EHI_005657 | ATCCAGGAAGTGAAGTTGAA | ATATCTTCGCATTGGTCTTG |
