# Supplementary material for: Overexpression of Differentially Expressed Genes Identified in Non-pathogenic and Pathogenic Entamoeba histolytica Clones Allow Identification of New Pathogenicity Factors Involved in Amoebic Liver Abscess Formation
Source: PLoS Pathog. 2016 Aug 30;12(8):e1005853. doi: 10.1371/journal.ppat.1005853 (PMC5004846; doi:10.1371/journal.ppat.1005853)
Supplement: S9 Table — (DOCX) [file ppat.1005853.s009.docx]

**S9 Table**  Oligonucleotides for gene amplification and for the analysis of overexpression in transfectants using qPCR.

|  |  | **Gene amplification** |  | **qRT-PCR** |  |
| --- | --- | --- | --- | --- | --- |
| **Gene name** | **Gene ID** | **Oligonucleotide sequence (5’)** | **Oligonucleotide sequence (3’)** | **Oligonucleotide sequence (5’)** | **Oligonucleotide sequence (3’)** |
| EhC2-3 (C2 domain containing protein) | EHI_015290 | GAGAGGTACCATGTCTGGAGGATATTACTCTCCTTTTG | GAGAGGATCCTCATTTCTTTTGTGGAGCTCCTTG | GATATCCACCACAACAAGG | GGTGGATAACCTGGATAAGC |
| EhMP8-2 (cell surface protease gp63) | EHI_042870 | GAGAGGTACCATGATTTTTTTGTTAGTCATTACAG | GAGAGGATCCTTAAAACATAACACAAAAGCTTTG | AAGATGGAGGAGGACGAGGT | CCCCAAGTGCCTCAAAATAA |
| EhRab7D (Rab family GTPase) | EHI_082070 | GAGAGGTACCATGGCAGGAAGACCAGCATTA | GAGAGGATCCTTAGCAACACCCTCCTTCT | CAACCGATACGTCAATAACC | TGCTCCATTAACAGTAACAGG |
| EhC2-5 (C2 domain containing protein) | EHI_059860 | GAGAGGTACCATGATCAAGATTGAATTAA | GAGAGGATCCTTAGAATGGGTTGTAGTAA | AAGTGGTTGATACTTGGTTAGG | GGAGCACCAACTGTTAAGC |
| EhC2-2 (C2 domain containing protein) | EHI_118130 | GAGAGGTACCATGTCCGTAGGATATAATTCA | GAGAGGATCCTTATTTTGGTGGTTGTTGTGG | AATGTTAAGCCATCAACTGG | AAGGATCAGAAGAACGAAGG |
| Hypothetical protein | EHI_075690 | GAGAGGTACCATGGTTAGTAAACTTGAACTA | GAGAGGATCCTTATTCATCTTTGTTATC | TTGCTAGATCAGCTGAAGG | CAACCATTCCTAATGAACC |
| EhRab7E (Rab family GTPase) | EHI_169280 | GAGAGGTACCATGTCAAATAAAAATCACAAG | GAGAGGATCCTCAACAACACCCTCCTT | ATTCTCATTGGTGATTCTGG | AATCAGCTCCAATAGTTGC |
| CAAX prenyl protease | EHI_075660 | GAGAGGTACCATGTTTCCATATTGGACAAG | GAGAGGATCCTTAATCACTTTTCATATCT | AGCAGAAGCAGCAGACC | AACCACATAGTTCCTTGTCC |
| Hypothetical protein | EHI_074080 | GAGAGGTACCATGTACTCAAAAGACGCTTAT | GAGAGGATCCCTAAAATCCTCCCATTGG | GGTTATCCACCACAACCAAT | CCATAGGACCTGACATTCCTC |
| EhRab7G (Rab family GTPase) | EHI_187090 | GAGAGGTACCATGCTAAAATTGATTTTAATTG | GAGAGGATCCTTAACAACAACCACTTTCAG | TTACTGCTATTGACACATGG | CTAACCTCGGTTGGCAGTCC |
| Hypothetical protein | EHI_048140 | GAGAGGTACCATGAAACAAAACACTAATT | GAGAGGATCCTTATTTTTTAGTTGTCTG | CACTGATTCTGGAAATGCTAC | TAGCACCTTTTGCTTTATCC |
| Hypothetical protein | EHI_058920 | GAGAGGTACCATGAATATAATATTTATTTG | GAGAGGATCCTTAAATCATTAATATAACTCCCA | TCACTCTGAAGATGGAACAA | TGGTGCACTGTCTATGTTATG |
| alcohol dehydrogenase | EHI_088020 | GAGAGGTACCATGAAAAATTTCACATA | GAGAGGATCCTTAGTAAATATCATTTAAG | TGGAGTTGAACAATGTTGG | TTGTGGAACCTCATAACACC |
| Hypothetical protein | EHI_151930 | GAGAGGTACCATGAACGCTATTAAACCTA | GAGAGGATCCTTATTTGTCTGAATTGTCT | CATAATGGTTATGGTGTTGC | CACACCATCTTCTTGAACG |
| Alcohol dehydrogenase 3 | EHI_160670 | GAGAGGTACCATGAAAATGGCAGGACAAG | GAGAGGATCCTTAGTAAATATCATTTAAG | TGGAGTTGAACAATGTTGG | TTGTGGAACCTCATAACACC |
| AIG1 family protein | EHI_180390 | GAGAGGTACCATGAAACAAACTAAG | GAGAGGATCCTTAATTTGACCACTT | CCAAAGTCAAAGAGTTCCCA | TGCCTTTGACAATACAACCA |
| Phosphoserine aminotransferase | EHI_026360 | GAGAGGTACCATGGAAAGGCAAAATATT | GAGAGGATCCTTAATGAGTATGTAATTGT | CTTGTTGCAGATATGTCTTCTG | TTGTGCACCTGCATAGATTA |
| 20 kDa antigen | EHI_056490 | GAGAGGTACCATGAGAGCACGTATGGTAG | GAGAGGATCCTTATTGTTGAGCAGCTGCT | ATGAGAGCACGTATGGTAGG | ATGAAGAAGGTCCACAACC |
| Actobindin | EHI_039020 | GAGAGGTACCATGGACGCTAAAGTACTT | GAGAGGATCCTTATTTAGCCTTGGCTTG | TGATAGAAATGAACTTCTTAGTGGAA | CCTTGGCTTGAATATCAGCA |
| Hypothetical protein | EHI_127670 | GAGAGGTACCATGTCCCTTAGTTTGTCA | GAGAGGATCCTCAGTATAAGCAAGTAACA | TGGGAGGAAGAGTGTGATGA | TTCCCGTTCTACATGACGAG |
| Hypothetical protein | EHI_144490 | GAGAGGTACCATGTCAAGAGTAGTAGTA | GAGAGGATCCCTAATATTGAATTTCTTTT | TATGCTTACATTCTGCGATG | TAAATACCATGGTGGGGTTA |
| Hypothetical protein | EHI_014170 | GAGAGGTACCATGAAATCATTATTAAAGAAG | GAGAGGATCCTTAATACTTAATCAATTCAAAACAAG | GCAGATCTTAATGGTGCTTCAA | CTACCACCAGCCTCACCAAG |
| Methionine gamma-lyase | EHI_144610 | GAGAGGTACCATGACTGCTCAAGATATTA | GAGAGGATCCTTAACAAAGCTCTAATG | ACATGCTTATCCAATCTTCC | CCAAGTCTAGAGTAGATATGACC |
| Leucine-rich repeat containing protein | EHI_073680 | GAGAGGTACCATGAATGAAAATAAAG | GAGAGGATCCTTAGTTTTTCTTTTTTAAT | TCAGAAAGTGCACCACAAGC | CCTCCTCCAAATCCTTGTCC |
